# Non-drug efflux function of ABCC5 promotes enzalutamide resistance in castration-resistant prostate cancer via upregulation of P65/AR-V7

**DOI:** 10.1038/s41420-022-00951-4

**Published:** 2022-05-03

**Authors:** Haojie Chen, Jia Luo, Shaojun Chen, Bowen Shi, Xiaocui Zheng, Haiying Ji, Xiaoqian Zhang, Yujia Yin, Kun Du, Jie Ding, Yongjiang Yu

**Affiliations:** 1 Department of Urology, School of Medicine, Xinhua Hospital Affiliated to Shanghai Jiao Tong University, Shanghai, 200092 P. R. China; 2 Department of Ophthalmology, Xinhua Hospital Affiliated to Shanghai Jiao Tong University School of Medicine, Shanghai, 200092 China; 3 Department of Obstetrics and Gynecology, Shanghai Jiao Tong University School of Medicine Xinhua Hospital, Shanghai, China; 4 Department of Anesthesiology and SICU, Xinhua Hospital, School of Medicine, Shanghai Jiao Tong University, Shanghai, 200092 China; 5 Department of Laboratory Medicine, Shanghai Jiao Tong University School of Medicine Xinhua Hospital, Shanghai, China

**Keywords:** Prostate cancer

## Abstract

Drug resistance is responsible for castration-resistant prostate cancer (CRPC)-associated mortality. While ATP binding cassette subfamily C member 5 (ABCC5) has been reported to regulate multiple drug resistance, its drug-efflux function may not be the main reason underlying resistance to enzalutamide, an androgen receptor inhibitor. Here, we aimed to determine whether the non-drug efflux function of ABCC5 affects enzalutamide resistance. The ABCC5 expression data in patients with prostate cancer (PCa) were retrieved from The Cancer Genome Atlas and Gene Expression Omnibus, and their correlation with disease prognosis was analyzed. Immunohistochemical staining was performed on a cohort of 80 patient samples. Proliferation of enzalutamide-resistant 22RV1 and C4-2B cells was investigated using CCK-8, EdU, and colony formation assays. The effect of ABCC5 silencing on enzalutamide resensitization was evaluated in vitro and in vivo. Functional assays indicated that ABCC5 depletion resensitized enzalutamide-resistant cells to inhibit cell growth and impeded xenograft tumor proliferation. Mechanistically, luciferase and ChIP assays confirmed that P65 regulated AR expression and activity by binding to its promoter, while ABCC5-mediated resistance effected by AR-V7 (one of the widely studied AR splicing variants that meditate AR antagonist resistance) upregulation could be reversed by P65 knockdown. Furthermore, activation of the NF-κB pathway reversed the effects of ABCC5 knockdown by extra AR-V7 expression. Thus, ABCC5 might be a novel target for enzalutamide-resistant CRPC treatment.

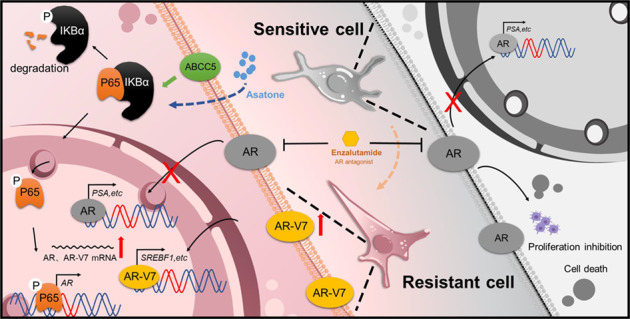

## Introduction

Prostate cancer (PCa) is the second leading cause of cancer-related death in American men, with approximately 248,530 newly diagnosed cases and 34,130 estimated deaths (accounting for 26% and 11% of total incident cases, respectively) in 2021 [1]. PCa progresses via two major stages to metastasis: androgen-dependent prostate cancer (ADPC) and castration-resistant prostate cancer (CRPC) [2]. Early-stage PCa can be controlled or even cured by androgen deprivation therapy (ADT), radical prostatectomy, and radiation therapy [3]. However, the CRPC stage is more difficult to treat, requiring the use of second-generation androgen receptor (AR) antagonists such as enzalutamide (also known as MDV3100) and chemotherapy [4]. As androgen and androgen receptors play essential roles in PCa development and progression, ADT is usually effective during the early period of treatment. This is inevitably accompanied by AR signaling reactivation, cancer relapse, and change of ADPC to CRPC [5, 6]. Several studies have revealed that the AR signaling pathway plays an essential role in the progression of CPRC [7]. Enzalutamide, a second-generation AR inhibitor, was approved as the first-line therapy for clinical therapy of CRPC [8, 9]. Unfortunately, most patients inevitably develop drug resistance and eventually die [10]. Although several mechanisms of enzalutamide resistance, including Wnt/β-catenin pathway activation, cholesterol biosynthesis, AR amplification, splicing variation, and activating mutations, have been reported [11–15], further investigations regarding the mechanisms underlying enzalutamide resistance are urgently required.

ATP-binding cassette subfamily C member 5 (ABCC5), also called MRP5 (multidrug resistance-associated protein 5), belongs to the C branch of the ATP-binding cassette transporter superfamily and has been reported to be a crucial factor involved in multiple drug resistance. ABCC5 uses the energy generated from ATP hydrolysis to exchange multiple substrates across both sides of the plasma membrane [16] and acts as a vital regulator of multidrug resistance [17–19]. ABCC5 has been reported to contribute to paclitaxel resistance by mediating the xenobiotic efflux of monophosphates of 6-mercaptopurine and 5-fluorouracil [20, 21]. Recent studies have indicated that ABCC5 expression increases abnormally in numerous malignant tumors, promoting tumor proliferation and metastasis. However, the molecular mechanism underlying the role of ABCC5 in CRPC enzalutamide resistance has not yet been investigated.

Recent studies have indicated that ABCC5 expression increases abnormally in numerous malignant tumors, promoting tumor proliferation and metastasis. However, the role of ABCC5 leading to enzalutamide resistance of CRPC has not yet been studied. In our present report, we found ABCC5 is a new potential therapeutic target that can alleviate enzalutamide resistance in patients with CRPC. However, ABCC5-mediated enzalutamide resistance did not cause by its classic drug efflux function, which enlightened us to deeper understand the underlying mechanism of hormone therapy and finally crack the drug resistance in prostate cancer.

## Results

### Database screening of ABCC5 related to enzalutamide-resistant prostate cancer

**Fig. 1:**
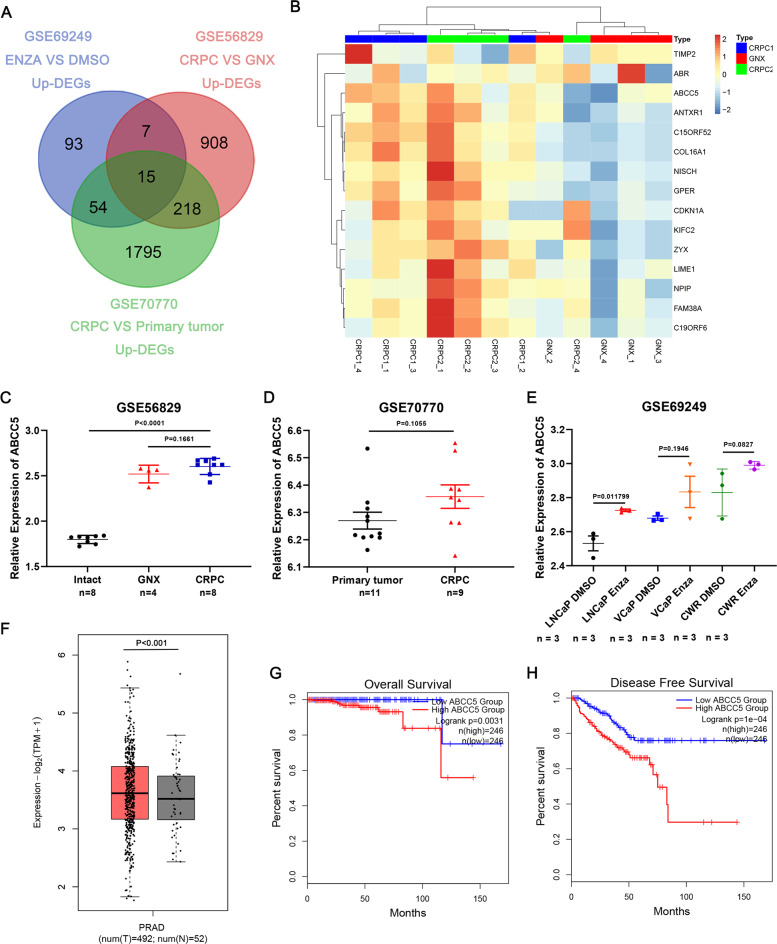
ABCC5 expression is related to enzalutamide-resistance PCa and CRPC. **A** The Venn diagram gives the overlapped genes of high expression related to enzalutamide-resistance and CRPC among the three GEO datasets above, including GSE69249 (enzalutamide treated vs. DMSO vehicle-treated LNCaP, VCaP, and CWR cell lines, top 10% up-regulated, *P* < 0.05), GSE56829 (CRPC vs. GNX, top 50% up-regulated, *P* < 0.05) and GSE70770 (CRPC vs. primary tumor, top 50% up-regulated, *P* < 0.05). **B** The heatmap with unsupervised hierarchical clustering of 15 selected genes mRNA expression in the GSE56829 dataset. The red represents for higher expression and the blue represents for lower expression. The expression of ABCC5 and that in comparison among VCaP xenograft tumors collected at different times which defined as intact, GNX, and CRPC groups in GSE56829 (**C**), and that of CRPC and primary tumor in the GSE70770 dataset (**D**) and that in comparison between enzalutamide treated and DMSO treated cells in GSE69249 (**E**). **F** The expression of ABCC5 in TCGA database, prostate tumor (Red) and normal (gray). Kaplan–Meier curves respectively for OS (**G**) and DFS (**H**), and of PCa patients with high versus low expression of ABCC5 in TCGA. *P < 0.05, and ****P < 0.0001.

To identify potential target genes in enzalutamide-resistant CRPC, we searched for drug resistance and CRPC related genes in the GEO database for further comprehensive analysis. We compared the different expression levels of genes between vehicle- and enzalutamide-treated LNCaP, VCaP, and CWR cell specimens from the GSE69249 dataset (enzalutamide responsive genes by genome-wide analysis) and found 169 differentially expressed genes (DEGs). We also identified 1148 and 2082 castration-resistant correlated genes in both the GSE56829 (VCaP xenograft tumors’ gene expression profiling study) dataset and GSE70770 dataset (expression profiling using array for prostate cancer stratification). The candidate genes from the three datasets were then integrated and 15 genes were selected (Fig. 1A). According to the heat map and hierarchical clustering, 15 DEGs independently clustered in the CRPC group (VCaP cell xenograft tumors collected 3–14 weeks after castration) and GNX group (VCaP cell xenograft tumors collected one day after castration) based on their differential expression levels in GSE56829 (Fig. 1B). Enrichment analysis of pathway and process suggested that candidate genes enriched in several bio-pathways, including small GTPase-mediated signal transduction, cell-substrate adhesion, and actin cytoskeleton organization (Supplemental Figure 1A), suggesting a drug resistance regulatory effect on hormone-refractory prostate cancer (Supplemental Figure 1B). To further select vital resistance-related genes, we analyzed TCGA, and 4 of 15 genes (*ABCC5, KIFC2, NPIPB3*, and *LIME1*) that were highly expressed and correlated with poor prognosis in malignancies were selected (Fig. 1F, Supplemental Figure 2A–C). Kaplan-Meier curves from TCGA indicated that the high expression of *ABCC5, KIFC2, NPIPB3*, and *LIME1* correlated with poor prognosis in terms of both overall survival (OS) and disease-free survival (DFS) (Fig. 1G and H, Supplemental Figure 2D-I).

Previous work has reported that ABCC5 over-expression associated with malignant metastasis of PCa. We found that ABCC5 expression was higher in the enzalutamide-treated cells than in the vehicle-treated cells in LNCaP, VCaP, and CWR cell lines in GSE69249, and that ABCC5 expression was higher in CRPC than in primary or GNX PCa (Fig. 1C–E). This suggested a positive correlation between ABCC5 and enzalutamide-resistant CRPC enzalutamide resistance.

### Expression of ABCC5 in prostate cancer and CRPC tissue

**Fig. 2:**
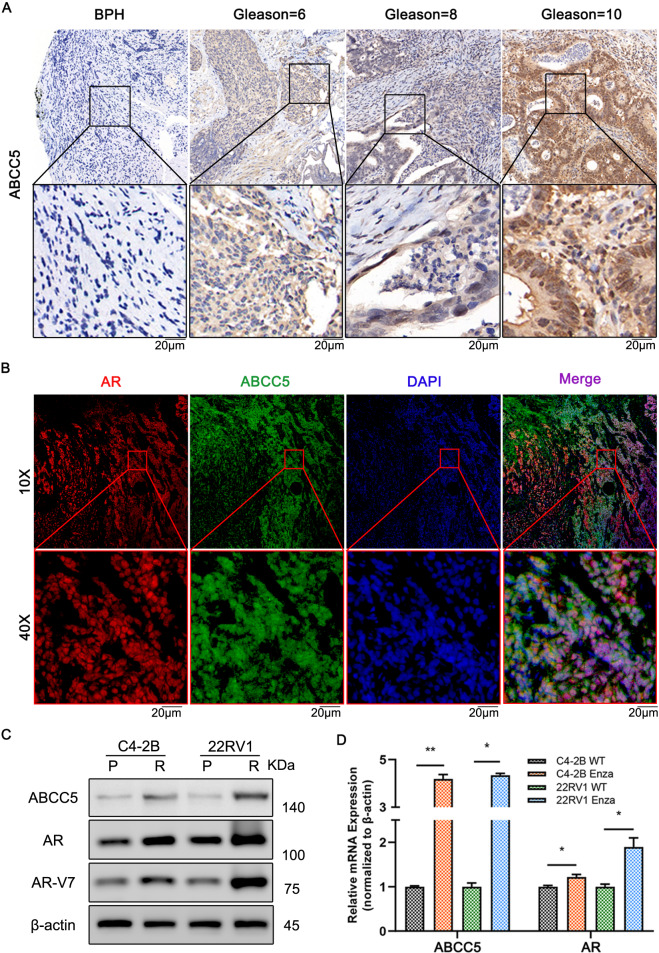
ABCC5 expression in prostate cancer patients. **A** Representative tissue staining images of ABCC5 expression in BPH and tumor tissues with several Gleason scores. Scale bars, 20 μm. **B** Immunofluorescence images of AR and ABCC5 localization in CRPC tissue. Scale bars, 20 μm. **C** PCa cells were subjected to the levels of selected proteins. **D** mRNA levels of AR and ABCC5 were measured by qRT-PCR. *P < 0.05, **P < 0.01, ***P < 0.001, ****P < 0.0001. P, parental cell; R, enzalutamide-resistant cell.

To study the correlations between ABCC5 and clinical enzalutamide-resistant CRPC progression at the protein level, we performed IHC staining on 80 samples (Supplementary Table 1), including 17 patients with BPH, 57 patients with primary PCa, and 6 patients with CRPC, and evaluated the correlation among ABCC5 expression, DFS and OS statistics from TCGA. Our analysis demonstrated that ABCC5 expression was significantly higher in PCa tissue than in BPH tissue (Fig. 2A), while stronger intensity of ABCC5-positive staining was associated with the high Gleason score. Analysis of PCa data from TCGA further confirmed that higher level of ABCC5 correlated significantly with worse DFS and OS. Furthermore, immunofluorescence of tumor tissue also confirmed the high expression of ABCC5 in CRPC (Fig. 2B).

### Protein and mRNA levels of ABCC5 were upregulated in resistant cells

To analyze the results of screening the GEO database mentioned above and to investigate the role of ABCC5 in drug resistance in vitro, we established two pairs of enzalutamide-resistant and enzalutamide-sensitive PCa cell lines, namely, C4-2B^Enza^ and C4-2B^WT^, and 22RV1^Enza^ and 22RV1^WT^, respectively, and evaluated their resistance by determining their IC50 for enzalutamide. The enzalutamide-resistant cell lines were less susceptible to enzalutamide than their parental cell lines, as they showed higher IC50 after enzalutamide treatment, confirming stronger resistance to enzalutamide (Supplementary Fig. 1C).

Despite being non-metastatic CRPC cells, C4-2B^Enza^ and 22RV1^Enza^ expressed significantly higher levels of both ABCC5 protein and mRNA than their sensitive counterparts. Furthermore, the protein and mRNA levels of AR were slightly elevated in resistant PCa (Fig. 2C and D), consistent with the results of our GEO database screening, which indicated that additional transcriptional mechanisms leading to elevation of ABCC5 level and activation of downstream pathways possibly contributed to the acquisition of enzalutamide resistance. AR-V7 (also known as AR3) level was high in resistant PCa cells. AR-V7 is one of the most widely studied AR splicing variants consisting only of exons 1–3 and a cryptic exon 3 (CE3), and generates a special transcript that encodes a truncated protein without the C-terminal ligand-binding domain (LBD). This truncated protein participates in ligand-independent transcriptional activity and contributes to the ligand-receptor complex-mediated nuclear translocation blocking effect of enzalutamide [22, 23]. Considering the inhibition of the AR pathway, ABCC5 might promote the proliferation of CRPC cells via alternative pathways to activate AR pre-mRNA splicing and generate variants, including AR-V7, ultimately leading to resistance to enzalutamide.

### ABCC5 inhibition restored enzalutamide sensitivity in vitro

**Fig. 3:**
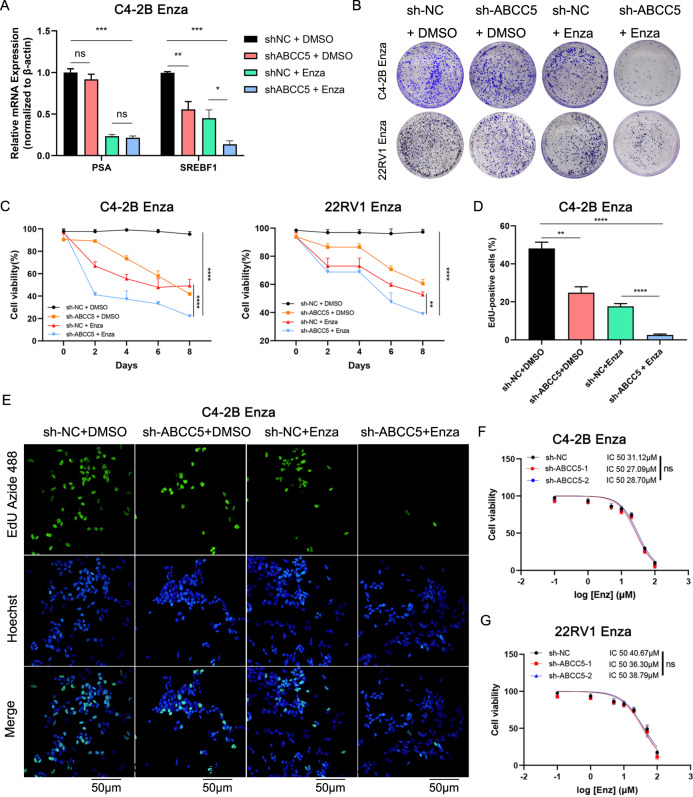
Targeting ABCC5 resensitizes the resistant PCa cells against enzalutamide in vitro. **A** Relative mRNA expression of AR and AR-V7 target genes (PSA, and SREBF1) were analyzed by qRT-PCR under the condition of C4-2B shNC and shABCC5 cells treated with or without 30 μM enzalutamide for 48 h. *P < 0.05, **P < 0.01, ***P < 0.001, n.s., not significant. **B** Colony formation assays were used in ABCC5-knockdown C4-2B^Enza^ and 22RV1^Enza^ cells. **C** C4-2B^Enza^ and 22RV1^Enza^ cells were cultured in 96-well plates for selected days followed with enzalutamide (30 µM) and harvested for CCK-8 assays. **D** The EdU positive cells were qualified to analyze relative percentage in C4-2B^Enza^ cells in three experiments. **E** Proliferation ability was detected by EdU assay to compare ABCC5 depletion and NC group with or without enzalutamide treatment in C4-2B^Enza^ cells. Scale bars, 50 μm. **F**–**G** Analysis of growth of ABCC5 knockdown C4-2B^Enza^ and 22RV1^Enza^ cells using CCK8. n.s., not significant.

To understand the mechanism via which ABCC5 controls drug resistance acquisition, the C4-2B^Enza^ and 22RV1^Enza^ cell lines were depleted of ABCC5 using lentivirus-encoded shRNA. qRT-PCR and western blotting were used to verify the efficiency of *ABCC5* knockdown after lentiviral infection. The results showed that the mRNA and protein levels of ABCC5 were significantly reduced in the C4-2B^Enza^ and 22RV1^Enza^ cell lines (Supplementary Fig. 4A and B). We investigated whether enzalutamide and ABCC5 ablation functioned synergistically to suppress the proliferation of drug-resistant cells. The combination of *ABCC5* knockdown and enzalutamide revealed a stronger suppressive effect on cell proliferation and growth in the colony formation assays (Fig. 3B), CCK8 (Fig. 3C), and EdU (Fig. 3D and E) in C4-2B^Enza^ and 22RV1^Enza^ cells. Moreover, the distinct mRNA expression levels of PSA and SREBF1 with or without enzalutamide treatment indicated that ABCC5 depletion exerted different effects on downstream targets regulation with enzalutamide burden (Fig. 3A). As a target of full-length AR, whether ABCC5 was knocked down or not, the mRNA level of PSA did not significantly alter, but it notably decreased with enzalutamide treatment. Furthermore, a recently reported AR-V7 specific target, SREBF1 [22], was remarkably down-regulated when ABCC5-ablation, and even lower when combined with enzalutamide-treatment, which indicating that ABCC5 may regulate drug resistance of PCa cells via AR-V7.

After enzalutamide treatment, the cells with ABCC5 depletion showed weaker proliferation than the control groups, suggesting that ABCC5 contributes to enzalutamide resistance. Furthermore, the IC50 for enzalutamide did not differ significantly between the control groups and shABCC5 groups, suggesting that the drug flux function of ABCC5 did not significantly affect enzalutamide resistance. In summary, while our results showed that ABCC5 repression can resensitize drug-resistant PCa cells toward enzalutamide, they also revealed a powerful synergy between ABCC5 depletion and enzalutamide treatment. In addition, the drug efflux function appeared to minimally affect the IC50 in enzalutamide-resistant cells with *ABCC5* knockdown (Fig. 3F and G).

### ABCC5 suppression facilitated enzalutamide potency in vivo

**Fig. 4:**
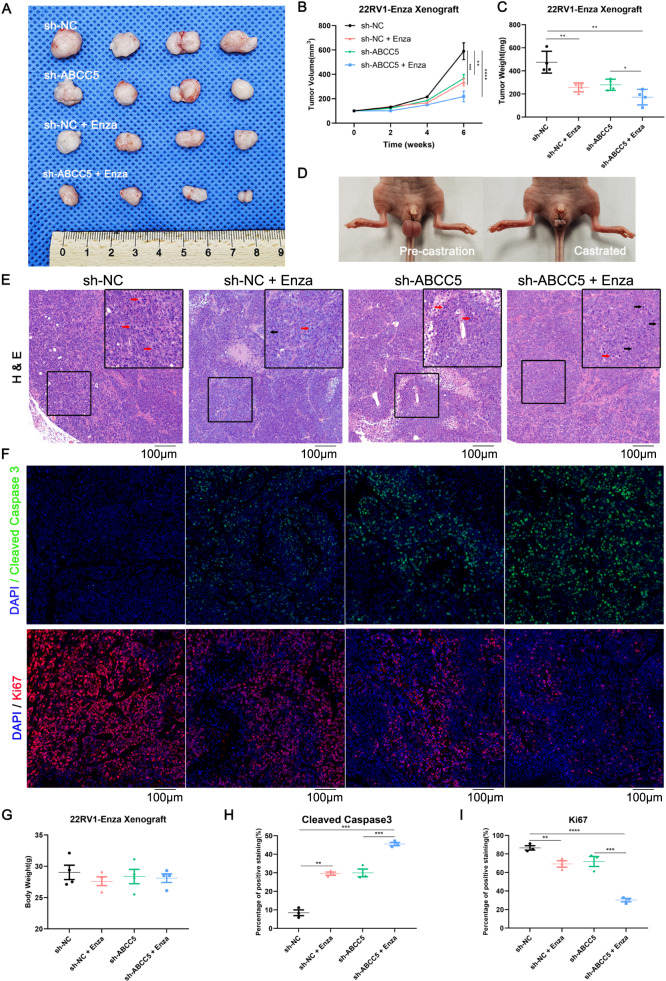
Knockdown of ABCC5 facilitates the efficacy of enzalutamide in vivo. **A** Representative images of the nude mice and xenograft tumors. 22RV1^Enza^ sh-NC cells or 22RV1^Enza^ sh-ABCC5 (2 × 10^6^cells/mouse) were suspended into Pre-castrated nude mice for 2 weeks followed by oral gavage enzalutamide (20 mg/kg) treatment. **B** Curves of Tumor growth for ABCC5-knockdown groups and controls with or without enzalutamide treatment are analyzed. The tumor volumes were measured every 2 weeks and expressed as mean ± SD. **C** Tumor weights of each variety treatment group were measured after being surgically dissected. **D** Nude mouse castration model representative images. **E** Representative results of tumor section by H&E staining. Red arrows represent mitotic cells, while black arrows represent apoptotic cells. Scale bars, 100 μm. **F** Representative images of IF staining for cleaved caspase 3 and Ki67. Scale bars, 100 μm. **G** Mice body weights were measured upon sacrifice. **H** Cleaved caspase 3–positive cells quantification in total numbers of cells. **I** Ki67-positive cells quantification in total numbers of cells. Data of at least three experiments as the mean ± SD. *P < 0.05, **P < 0.01, ***P < 0.001, ****P < 0.0001.

To assess the resensitization of PCa cells to enzalutamide by targeting ABCC5 in vivo, xenografted 22RV1^Enza^ cells were subjected to the combination treatment (*ABCC5* knockdown and enzalutamide treatment) in nude mouse that were surgically castrated one week before injection (Fig. 4D). Tumor volumes decreased in the shABCC5 groups (Fig. 4A and B), whereas the shABCC5 plus enzalutamide treatment group showed a synergistic effect with dramatic tumor repression, indicating that the *ABCC5* knockdown overcame enzalutamide resistance and restored the sensitivity of resistant cells to enzalutamide. Although both tumor net weight and size decreased after *ABCC5* knockdown or enzalutamide treatment alone, ABCC5 depletion combined with enzalutamide treatment showed a more significant inhibitory effect on xenograft tumor proliferation than enzalutamide alone (Fig. 4C). Moreover, no significant cumulative effect on the body weight of the castrated tumor-bearing nude mice was observed in vivo (Fig. 4G), indicating that the combination therapy exerted negligible negative effect on the normal survival state. H-E staining of the control group with no drug treatment revealed more active mitotic cells comparing with drug-treated group. In contrast, the tumors derived from ABCC5-ablated cells with enzalutamide treatment exhibited more apoptotic bodies and pyknotic nuclei comparing with the control groups (Fig. 4E). To determine the combined effect of enzalutamide treatment and ABCC5 depletion on inhibition of tumor proliferation and promotion of apoptosis, immunofluorescence staining for Ki67 and cleaved caspase 3 was performed on xenograft tumor slices (Fig. 4F). Increase in the number of cleaved caspase 3-positive cells, along with reduction in Ki67-positive cells, was observed after the combination treatment, suggesting strong induction of apoptosis and inhibition of proliferation (Fig. 4H and I). Consistent with the results of cell-based studies, these observations suggested that ABCC5 ablation acts synergistically with enzalutamide treatment, might become a promising therapeutic strategy enzalutamide-resistant treatment.

### ABCC5‐related signaling pathways identification

To understand the mechanism responsible for the positive correlation between ABCC5 and AR-V7 expression during enzalutamide treatment, we investigated whether the AR pathway was regulated by ABCC5 in the resistant cells. As mentioned above, the elevated ABCC5 and AR protein levels correlated with drug resistance in enzalutamide-resistant cells (Fig. 2C and D). However, low PSA mRNA level was clearly suggestive of an inactive AR pathway (Fig. 3A), indicating that high level of ABCC5 could not be the result of AR pathway activation. Surprisingly, the alteration in ABCC5 protein level was consistent with AR-V7 elevation, which has been reported as the most frequently expressed AR variant functionally relevant for CRPC development [24]. Hence, we attempted to uncover the potential mechanisms underlying the ABCC5-activated splicing variation of AR to AR-V7 in enzalutamide-resistant cells. AR splice variants participate in the acquisition of enzalutamide resistance [22, 25]. Increase in the level of AR-V7 with a truncated C-terminus, which could not be inhibited by the ligand-binding domain inhibitor, enzalutamide, caused continuous activation of AR signals, resulting in prostate cancer resistance to enzalutamide.

**Fig. 5:**
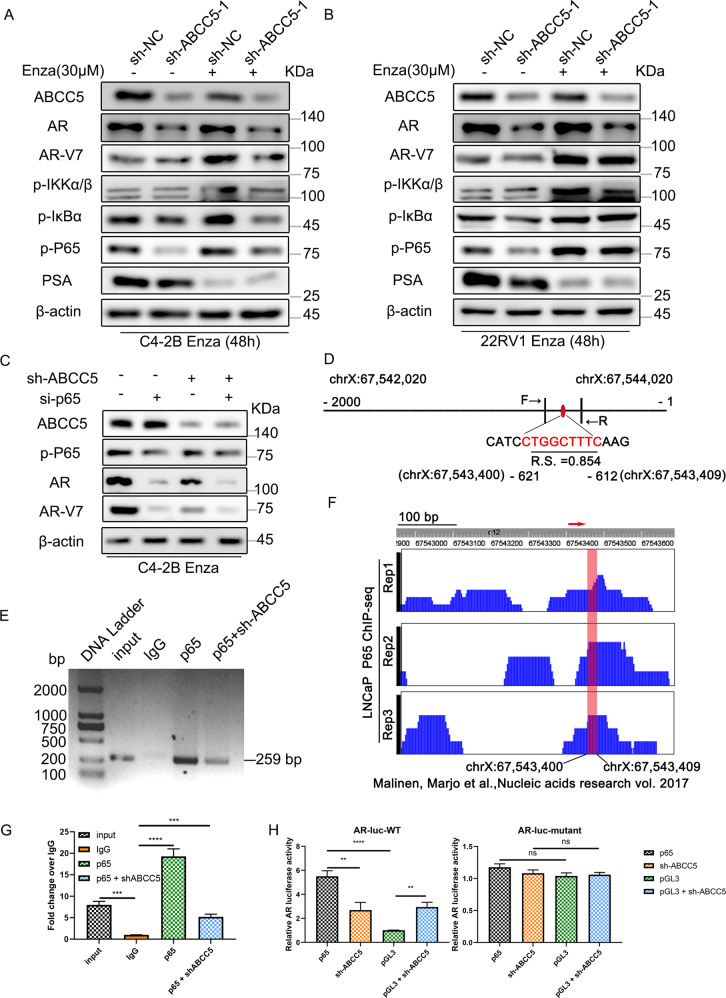
Identification of ABCC5‐related signaling pathways. **A**, **B** DMSO or enzalutamide was treated in C4-2B^Enza^ and 22RV1^Enza^ cells with ABCC5 depletion or control groups, then cells were subjected to western blotting analysis. **C** Targeting ABCC5 regulates the protein level of p-P65, which in turn influences the expression of AR and AR-V7. **D** We scanned the promoter of AR using the JASPAR website and predicted one potential binding site (CTGGCTTTC) for P65. Relative score (RS) for the motifs was 0.854. **E** Primer F and primer R were designed to amplify the CTGGCTTTC motif. Results of ChIP DNA electrophoresis confirmed that P65 can bind to the motif in C4-2B^Enza^ cells. **F** ChIP‐seq results from Malinen, Marjo et al. showed the binding site dominion of P65 in the AR gene promoter region in LNCaP cells (indicated in red). **G** ChIP-quantitative PCR results analysis showed that P65 bound to the AR gene promoter region; fold change of input and P65 expression relative to IgG; IgG, chromatin fragments pulled down by anti-IgG antibody; P65 + shABCC5, chromatin fragments pulled down by anti-P65 antibody in ABCC5 depletion PCa cells. **H** Dual luciferase reporter assay showed that ABCC5 knockdown partially suppressed the activity of the wild-type AR promoter. Moreover, the ABCC5 knockdown had no effect on the activity of mutant AR promoter luciferase reporter in C4-2B^Enza^ cells. Data of at least three experiments as the mean ± SD. **P < 0.01, ***P < 0.001, ****P < 0.0001, n.s., not significant. RS relative score.

A recent study revealed that the activation of NF-κB signaling promotes AR-V7 to replace full-length androgen receptor (AR-FL) and leads to the progression of CRPC [26]. We found that after treatment with enzalutamide, the expression of AR and AR-V7 in the control group increased significantly, and that the NF-ΚB pathway was also activated. The expression levels of the NF-κB signaling pathway molecules and AR and AR-V7 decreased when *ABCC5* was knocked down (Fig. 5A and B); thus, we speculated that ABCC5 may regulate AR expression via the NF-κB signaling pathway.

To verify whether the NF-κB subunit, P65 (also known as RelA), mediates the pro-resistant effect of ABCC5 in enzalutamide resistance by translocating into the nucleus and function as a transcription factor, we knocked down P65 in ABCC5-depleted PCa cells. We found that suppression of ABCC5 was followed by a considerable blockade of phosphorylated P65, AR, and AR-V7 expression. We then inhibited P65 activity using small interfering RNA with and without ABCC5 depletion and found that AR species were significantly more downregulated when ABCC5 and P65 were interfered with at the same time than when they were depleted individually. Importantly, AR and AR-V7 expression were significantly lower when P65 inhibited alone than that in the ABCC5 non-knockdown group, which suggests that the expression of P65 and its phosphorylated form is required for the regulation of AR-V7 (Fig. 5C).

Based on the results of previous studies showing that P65 (RelA) can specifically regulate the expression of AR-V7, we hypothesized that the drug-resistant effect of ABCC5 is mediated by P65-mediated regulation of the expression of AR and its splicing variants. To investigate the role of NF-κB P65 in the transcriptional regulation of AR via ABCC5, we searched the JASPAR (http://jaspar.genereg.net) to find putative binding sites in the AR promoter. Sequence analysis revealed one putative binding site (Supplementary Fig. 3A). JASPAR analysis gave potential binding sites a quantitative score, based on the probability of observing each nucleotide at each position of the binding motif compared to that in consensus sequences of known binding sites. The sequence, location in the promoter before the transcription starting location, and normalized relative score were CTGGCTTTC, −621 relative to −612 bp, and 0.854, respectively (Fig. 5D). We performed ChIP assay to verify the P65 binding with the AR promoter region (Fig. 5E and G). We then detected the AR promoter activity in wild-type C4-2B PCa cells. Luciferase reporter assay in C4‐2B cells which co-transfection with full‐length AR or binding region mutant, resulted in different AR promoter activity, with pGL‐3 as a control vector (Fig. 5H). Notably, ABCC5 depletion significantly affected AR promoter activity, blocking the inductive effect of P65. Moreover, the P65 ChIP-seq results from Malinen et al. [27] also showed a peak signal in the AR promoter region in this domain (Fig. 5F).

Our experimental results also confirmed that not only did the protein level of phosphorylated P65 decrease, but the levels of AR and AR-V7 were also significantly reduced after ABCC5 downregulation. Therefore, ABCC5 enhances the expression of phosphorylated P65, which in turn activates transcription by binding to the downstream AR promoter to enhance AR protein expression and pre-mRNA splicing, ultimately promoting enzalutamide resistance in prostate cancer.

**Fig. 6:**
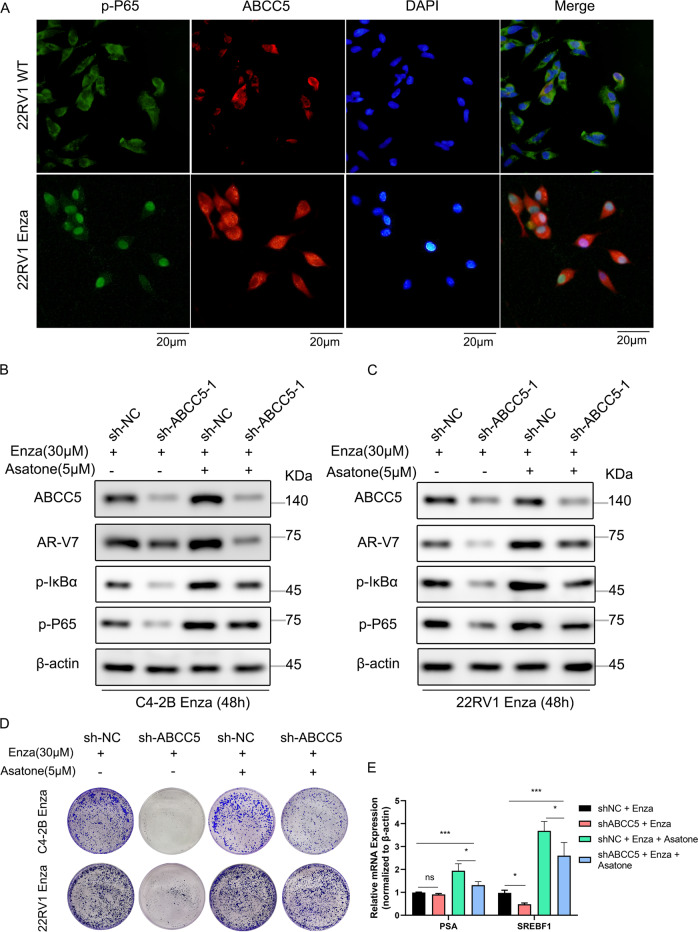
NF-κB agonist Asatone rescues the function of promoting enzalutamide resistance in ABCC5-knockdown cells. **A** Representative images of confocal microscopy immunofluorescence staining for ABCC5 and p-P65 co-localization in 22RV1^WT^ and 22RV1^Enza^ cells. Scale bars, 20 μm. The levels of ABCC5, AR-V7, phosphorylated IKBα, and phosphorylated P65 were detected by Western blotting in ABCC5-knockdown and control C4-2B^Enza^ (**B**) and 22RV1^Enza^ (**C**) cells combined with or without Asatone (5 µM) in enzalutamide treatment. **D** ABCC5-knockdown and control C4-2B^Enza^ and 22RV1^Enza^ cells with or without Asatone in enzalutamide treatment for colony formation assays construction. **E** Relative mRNA expression of AR and AR-V7 target genes (PSA, and SREBF1) were analyzed by qRT-PCR under the condition of C4-2B shNC and shABCC5 cells treated with or without 5 μM Asatone accompanied by 30 μM enzalutamide for 48 h. *P < 0.05, ***P < 0.001, n.s., not significant.

In addition, to investigate the relationship between ABCC5 and p-P65, ABCC5 and p-P65 co-localization was analyzed in 22RV1^WT^ and 22RV1^Enza^ cells via immunofluorescence. Interestingly, the results showed that ABCC5 was mainly expressed in the cytoplasm and was rarely detected in the nucleus in wild-type cells, whereas p-P65 did not show any signs of nuclear translocation. Furthermore, in 22RV1^Enza^ cells, accompanied by an increase in ABCC5 expression, p-P65 translocated to the nucleus (Fig. 6A). A similar result was observed in C4-2B cells (Supplementary Fig. 3B).

Furthermore, results of the fractionation assay showed that in drug-resistant cells, the expression of p-P65 in the nucleus accounted for a significantly higher proportion of the overall expression than that in the cytoplasm, suggesting nuclear translocation of P65. Although the overall expression of ABCC5 in drug-resistant cells increased significantly, its expression in the nucleus did not increase, indicating that evidence for its nuclear translocation was not sufficient (Supplementary Fig. 4C).

### ABCC5 facilitated enzalutamide resistance via the NF-κB/AR-V7 signaling pathway

To further probe the connection between ABCC5 and the NF-κB P65 signaling pathway in the context of AR-V7 activation, we performed a protein expression assay to determine whether ABCC5 can promote AR-V7 via the NF-κB pathway in PCa cells. We found that *ABCC5* knockdown reduced the levels of phosphorylated NF-κB P65 and AR-V7. Moreover, ABCC5 ablation reduced the levels of the phosphorylated forms of IKBα and NF-κB P65 (Fig. 6B and C). Our results suggested that ABCC5 might function as a potential activator of the NF-κB signaling pathway and enhance the AR-V7 protein level to promote enzalutamide resistance in PCa.

To investigate whether ABCC5 regulates PCa cells in an NF-κB pathway-dependent manner, PCa cells with *ABCC5* knockdown were treated with Asatone, an agonist of NF-κB [28], to rescue NF-κB pathway-specific activation in C4-2B^Enza^ and 22RV1^Enza^ cells. We found that Asatone (5 µM) reversed *ABCC5* knockdown-induced AR-V7 repression via the activation of the NF-κB pathway, which occurred simultaneously with the activation of p-IKBα and p-P65. Interestingly, activation of the NF-κB pathway increased the expression of AR-V7 in vitro, which enhanced enzalutamide resistance in ABCC5-ablated cells.

For further confirmation, ABCC5-ablated PCa cells were treated with Asatone (5 µM) for the colony formation assay. The results showed that treatment with Asatone in stable ABCC5-depleted enzalutamide-resistant PCa cells rescued *ABCC5* knockdown-induced resensitization toward enzalutamide and markedly promoted the proliferation ability of the ABCC5-depleted group compared to that of the control group (Fig. 6D). The activation of downstream target SREBF1 rather than PSA by Asatone treatment indicated that the ability of Asatone to rescue the drug resistance on *ABCC5* knockdown cells relied on specific activation of AR-V7 rather than full-length AR (Fig. 6E). Overall, all the results indicated that ABCC5 promoted enzalutamide resistance of PCa cells via the NF-κB P65/AR-V7 pathway.

## Discussion

Enzalutamide resistance has been reported to be related to high level of AR expression. In PCa, targeting of AR signaling has been successfully used as the main therapy in the early period of the disease. However, previous reports have revealed that the tumors mostly relapse and advance to CRPC even after ADT followed by enzalutamide treatment [29], which indicates that elevated expression of AR alone is not sufficient for drug resistance in ADT. For example, enzalutamide-sensitive LNCaP cells with exogenous AR-FL overexpression but without AR-V7 expression remained enzalutamide-sensitive in vitro and in vivo [30]. In addition, our present work has also shown that in C4-2B cells, in addition to the significant increase in endogenous AR-FL level, AR-V7 expression increased the level of total normalized AR protein to more than ten-fold than that in parental cells after long-term treatment with enzalutamide. In fact, a corollary of these findings is that enzalutamide resistance in PCa involves both elevated expression of AR-FL and detectable expression of AR-V7.

**Fig. 7:**
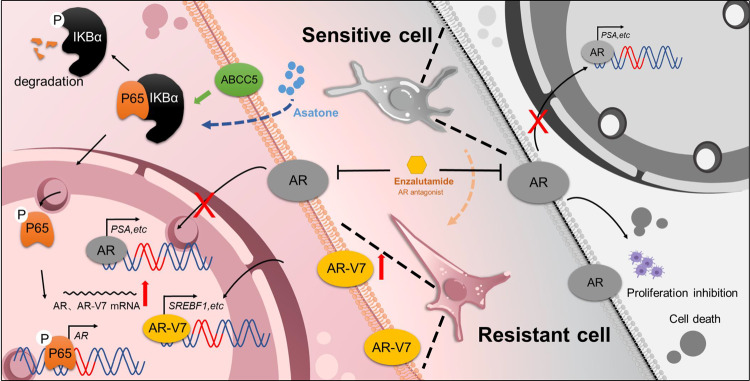
Schematic diagrams illustrating the role of ABCC5. The long-term burden of enzalutamide treatment induces ABCC5 high expression. Elevated ABCC5 promoted P65 phosphorylation and promotes AR-antagonist resistance via AR splicing variant 7 generation.

Next, we investigated the mechanism underlying the increase in AR-V7 expression during the progression of PCa. Our study demonstrates that level of AR-V7 increased abnormally with increase in ABCC5 expression via the activation of the NF-κB pathway in CPRC tissue and enzalutamide-resistant PCa cells. As ABCC5 is involved in multiple drug resistance because of its classic drug efflux function, increase in ABCC5 expression may reduce cellular intake of toxic drugs, including enzalutamide [19, 31–33]. However, this hypothesis was negated by the similar IC50 of *ABCC5* knocked down PCa cells and control groups, indicating that the efflux function of ABCC5 does not play a critical role in enzalutamide resistance in PCa. Although the underlying mechanism requires further elucidation, our study has demonstrated that ABCC5 functions as an activation co-regulator of the NF-κB subunit P65 and activates the AR signaling pathway by binding to the AR promoter region (Fig. 7).

It is noteworthy that copy number gain in the AR locus has been reported as one of the major mechanisms underlying ADT resistance in PCa, and that AR amplification occurs in the majority of CRPC and metastatic CRPC [34, 35]. In agreement with these observations, our present study indicated a positive correlation between ABCC5 and AR in advanced PCa. Elevated levels of ABCC5 promoted P65 expression and phosphorylation, which confirmed the transcriptional activity of the ABCC5-P65 co-activator at the AR promoter binding site Although the ABCC5-P65 co-activator could not reactivate the AR signaling pathway and its downstream targets like PSA which was inhibited by enzalutamide, in other words, the full-length AR signaling pathway still remained silent, the ABCC5-mediated co-activation provides a basic mechanism for the elevation in AR mRNA expression in most, but not all, advanced PCa, whereas it explicated the increase in AR-V7 expression proportionally with AR-FL in CRPC. This transcriptional increase in total AR pre-mRNA may be sufficient to increase the mRNA and protein expression of AR-V7 owning a cryptic exon 3 (CE3), which circumvents the enzalutamide-mediated blockage of nuclear translocation of the ligand-receptor complex and activate AR-V7-relied downstream targets, like SREBF1, to maintain the proliferation activity of PCa cells, increases drug resistance [36]. A recently published finding has reported that ABCC5 exerted a protumor effect to promote the phosphorylation of AR at Ser81 by CDK1 and activated the AR downstream target genes and promoted the malignant progression and enzalutamide resistance of prostate cancer [37], although it was unclear if ABCC5 transcriptionally promoted the AR expression and how it improved the drug resistance. Our findings focused on the ABCC5-mediated enzalutamide resistance and indicated that ABCC5 regulates the activity of NF-κB-p65 and generation of AR transcripts, further explaining the potential AR upstream mechanism of ABCC5-mediated enzalutamide resistance, indicating that future development of specific ABCC5 inhibitors may be a promising strategy to target prostate cancer AR-antagonist resistance.

## Materials and methods

### Analysis of data from the Gene Expression Omnibus (GEO) and The Cancer Genome Atlas (TCGA) databases

Three GEO datasets, including GSE69249 (including three enzalutamide-treated LNCaP, VCaP, and CWR samples, together with three dimethyl sulfoxide-treated samples), GSE56829 (consisting of eight CRPC samples and four GNX samples. CRPC: VCaP cell xenograft tumors collected 3-14 weeks after castration; GNX: VCaP cell xenograft tumors collected one day after castration), and GSE70770 (including nine CRPC samples and 11 robotic radical prostatectomy primary tumor tissue samples of similar age) were analyzed using the GEO2R Analysis tool (https://www.ncbi.nlm.nih.gov/geo/geo2r/). The top 10% upregulated genes with P < 0.05 in GSE69249 and the top 50% with *P* < 0.05 in GSE56829 and GSE70770 were selected for further investigation. Data from TCGA (https://gdc.cancer.gov/) were also collected to analyze the correlation between gene expression and information on clinical survival.

### Human tissue samples

In total, 80 paraffin-embedded samples from 17 patients with benign prostatic hyperplasia (BPH) patients, 57 with primary PCa, and 6 with CRPC were obtained with the written consent of patients who underwent radical prostatectomy or transurethral resection prostate (TURP) at Xinhua Hospital affiliated to the Shanghai Jiao Tong University School of Medicine. All samples were diagnosed with prostate cancer or benign prostatic hyperplasia by two independent pathologists. Ethical approval was obtained from the Xinhua Hospital’s Committees for Ethical Review of Research Involving Human Subjects.

### Immunohistochemistry (IHC)

Tumor tissue blocks were cut into 4-µm-thick sections for IHC staining. H_2_O_2_ (3%) was used to block the endogenous peroxidase activity. Relative antibodies and dilutions were shown in the supplemental material. The immunohistochemical reactions were developed with diaminobenzidine, and Harris hematoxylin was used to counterstain the sections.

### Total RNA isolation and quantitative reverse transcription-polymerase chain reaction (qRT-PCR)

Total RNA was isolated using TRIzol reagent (Thermo Fisher Scientific, Waltham, USA, 15596018) following the manufacturer’s protocol. cDNA was synthesized using the PrimeScript RT reagent kit (Takara Biomedical Technology, Beijing, China, RR055), and qRT-PCR was performed using Hieff qPCR SYBR Green master mix (Yeasen Biotech, Shanghai, China, 11202ES03). Relative gene expression in each sample was analyzed using the threshold cycle (CT) method. Each reaction was performed in triplicate. *β*-*actin* was used as a reference gene for other mRNAs. Primers for qRT-PCR were listed in the supplementary material.

### Western blotting

The cells were prepared using radioimmunoprecipitaion assay buffer (Thermo Fisher Scientific, 89900) supplemented with Halt protease and phosphatase (Thermo Fisher Scientific, 78440), and protein concentration was determined using the bicinchoninic acid protein assay kit (Beyotime Biotechnology, Shanghai, China, P0012). The proteins were separated via 10% sodium dodecyl sulfate-polyacrylamide gel electrophoresis and transferred onto polyvinylidene difluoride membranes. Freshly prepared 5% non-fat milk in 1X TBST buffer (25 mM Tris, pH 7.5, 137 mM NaCl, 2.7 mM KCl, and 1 ml Tween 20) was used for blocking for 2 h at room temperature. The blots were then incubated with primary antibodies overnight at 4 °C. Next, the membranes were washed thrice with 1X TBST buffer for 5 min each time and then incubated with a horseradish peroxidase-labeled secondary antibody. The membranes were visualized via chemiluminescence using enhanced chemiluminescence (ECL) reagents and an ECL Plus detection system. All the primary antibodies used in this study with dilutions and vendor details were shown in the supplemental material.

### Chromatin immunoprecipitaion (ChIP) and dual luciferase assay

More than 1 × 10^7^ cells C4‐2B^Enza^ cells were crosslinked using 4% formaldehyde mixed with Roswell Park Memorial Institute (RPMI) 1640 medium in a Petri dish (final concentration, 1%), followed by incubation at 37 °C in a culture chamber for 15 min. Then 5 M glycine solution was added to a final concentration of 0.125 M and incubated for 15 min at room temperature. Phosphate-buffered saline containing 1 M phenylmethylsulfonyl fluoride was used to wash the cells twice, followed by scraping of the cells in a 1.5 ml Eppendorf tube. ChIP was performed using the ChIP assay kit (Beyotime Biotechnology, P2078). Generally speaking, the cells were sonicated 10 seconds each time, a total of 4 times with a 2 mm Ultrasound head (50 W). RT-qPCR was performed after purifying the DNA fragments. The input control was extracted from the supernatants of sonicated lysates. PCR was performed to demonstrate the binding region of P65 in AR enhancer domain, and the primer sequences are shown in the supporting information. The expected region for ChIP primers was 259 bp.

Synthetic reporter plasmids, pGL3‐AR-WT and pGL3‐AR‐mutant (shown in Supporting Information), were used in the dual luciferase assay. The reporter plasmids were co-transfected with phRL‐TK as an internal reference. After 48 h of transfection, dual luciferase assays were performed using a kit produced by Yeasen Biotech (Shanghai, China, 11402ES60).

### Cell culture and reagents

The human prostate cancer cell line, 22RV1, was purchased from the Cell Bank of the Chinese Academy of Science (Shanghai, China). C4-2B cells were kindly provided by Dr. Xun Shangguan of Renji Hospital, School of Medicine, Shanghai Jiao Tong University. Short tandem repeat (STR) DNA profiling was performed to verify the cell lines mentioned above. Two drug-resistant cell lines (C4-2B^Enza^ and 22RV1^Enza^) were established by exposing parental C4-2B and 22RV1 cells to increasing concentrations of 5–40 µM enzalutamide for more than 12 months, as described previously [36]. Wild-type PCa cells were cultured in RPMI 1640 medium (Cytiva, Shanghai, China, SH30809.01) supplemented with 10% fetal bovine serum (Thermo Fisher Scientific, 26140079), while the resistant cells were cultured in RPMI 1640 medium with 10% charcoal-stripped fetal bovine serum (Thermo Fisher Scientific, 12676029). Two enzalutamide-resistant cells were cultured in a previously described medium containing 20 µM enzalutamide to maintain drug resistance. All cell lines were cultured in the presence of 100 units/ml penicillin (Thermo Fisher Scientific, 15140122) and 100 units/ml streptomycin (Thermo Fisher Scientific, 15240062) and incubated at 37 °C in a humidified 5% CO_2_ chamber. Enzalutamide was obtained from Selleck Chemicals (Shanghai, China, S1250). Asatone was obtained from MedChem Express (USA, HY-N6826).

### Cell viability assay

Cellular proliferation was detected using the cell counting kit-8 (CCK-8) assay (APExBIO Technology, Houston, USA, K1018). The cells were seeded in 96-well plates at a density of 1 × 10^3^ cells per well, cultured for 24 h, and then treated with 30 µM enzalutamide. After incubation with the CCK-8 testing reagent for 30 min, cell viability was measured at 0, 2, 4, 6, 8 days after seeding.

### Colony formation assay

Cells (5 × 10^3^ cells /well) were plated on 6 cm dishes plates and cultured in 1640 medium alone or medium containing 30 µM enzalutamide for 14 days (with or without 5 µM Asatone treatment). After culturing, the cells were fixed using 4% paraformaldehyde and stained with 1% crystal violet solution for 20 min, followed by the colony formation calculation using imageJ software.

### In vivo mouse model xenograft experiments

All procedures involving animals were approved by the Xinhua Hospital Animal Ethics Committee, which is in agreement with the Guidance for Care and Use of Laboratory Animals. Male BALB/c nude mice (4 weeks old) were provided by the Animal Experiment Center (Super-B&K Laboratory Animal Corp. Ltd, Shanghai, China) and maintained at the Xinhua Hospital Laboratory’s animal specific pathogen-free (SPF) barrier facilities. For the xenograft model, 22RV1^Enza^ cells (2 × 10^6^cells/mouse) were suspended in 200 µl culture medium with 50% Matrigel matrix (Corning Life Sciences, NY, USA, 354248) and subcutaneously injected into the dorsal flank of castrated BALB/C mice. The xenografted mice were randomly divided into two groups and treated with 200 µl vehicle control or enzalutamide (20 mg/kg) via oral gavage. Four mice were included in each group.

Tumor volumes were measured using Vernier calipers after every 7 days and analyzed using the formula *V(mm*^*3*^*)* = *L(mm)* × *W(mm)* × *T (mm)* × π/6 (where *V* represents volume, *L* represents length, *W* represents width, and *T* represents thickness). The mice were euthanized eight weeks after injection, and the tumors were surgically dissected. Xenograft tumors were fixed using 4% paraformaldehyde, embedded in paraffin, sectioned to 5 µm, and stained using conventional hematoxylin-eosin (H-E) staining for histological analysis. Immunofluorescence staining was performed using the MOM kit (Vector Laboratories, Shenzhen, China, PK-2200) [38]. Tumor weight and body weight were also recorded.

### Statistical analysis

All experiments were repeated at least three times. Paired t-tests or one-way analysis of variance was used to compare the control and experimental groups. Three independent samples were acquired to perform statistical analyses. Statistical significance was set at *P* ≤ 0.05. All data analyses were performed using the IBM SPSS Statistics (Version 25.0; IBM Corp.).

## Supplementary information

Supplementary Material

Supplementary WB original scan

## Data Availability

The datasets supporting the conclusions of this study are available from the corresponding author upon request.
