# Supplementary material for: Non-drug efflux function of ABCC5 promotes enzalutamide resistance in castration-resistant prostate cancer via upregulation of P65/AR-V7
Source: Cell Death Discov. 2022 May 3;8:241. doi: 10.1038/s41420-022-00951-4 (PMC9065095; doi:10.1038/s41420-022-00951-4)
Supplement: Supplementary file 1 — Supplementary Material [file 41420_2022_951_MOESM1_ESM.docx]

Non-drug efflux function of ABCC5 promotes enzalutamide resistance in castration-resistant prostate cancer via upregulation of P65/AR-V7

Haojie Chen^1†^, Jia Luo^2†^, Shaojun Chen^1^, Bowen Shi^1^, Xiaocui Zheng^3^, Haiying Ji^4^, Xiaoqian Zhang^3^, Yujia Yin^3^, Kun Du^5*^, Jie Ding^1*^, Yongjiang Yu^1*^

**Supplemental Material**

**Supplemental Figure Legends**

Supplemental Figure 1

(A) Summary of the 15 genes enrichment analysis in DisGeNET. (B) The top-level Gene Ontology biological processes (C) The half-maximal inhibitory concentration (IC50) of wild-type and enzalutamide resistant prostate cancer cell lines.

Supplemental Figure 2

(A-C) The expression of KIFC2, LIME1 and NPIPB3 in TCGA database, prostate tumor (Red) and normal (grey). (D-I) The overall survival (OS) and disease-free survival (DFS) Kaplan-Meier curves of KIFC2, LIME1, NPIPB3 from the TCGA database.

Supplemental Figure 3

(A) Sequence analysis revealed putative p65 binding sites in the AR promoter binding site. (B) Representative images of confocal microscopy immunofluorescence staining for ABCC5 and p-P65 co-localization in C4-2BWT and C4-2BEnza cells. Scale bars, 20 μm.

Supplemental Figure 4

(A-B) C4-2BEnza and 22RV1Enza cells were cultured with sh-NC or sh-ABCC5 lentivirus, followed by Western blotting analysis (A) and qRT-PCR (B). (C) Subcellular localization of ABCC5 and p-P65 in C4-2B cells. Cytosolic (C) and Nuclear (N) fractions were prepared from C4-2B wild-type and enzalutamide resistant cells, followed by immunoblotting analysis with antibodies as indicated.

Supplemental Figure 5

(A) Representative images of tunel staining for checking cell apoptosis in tumor tissues. Scale bars, 20 μm.

**Supplemental Figure 1**


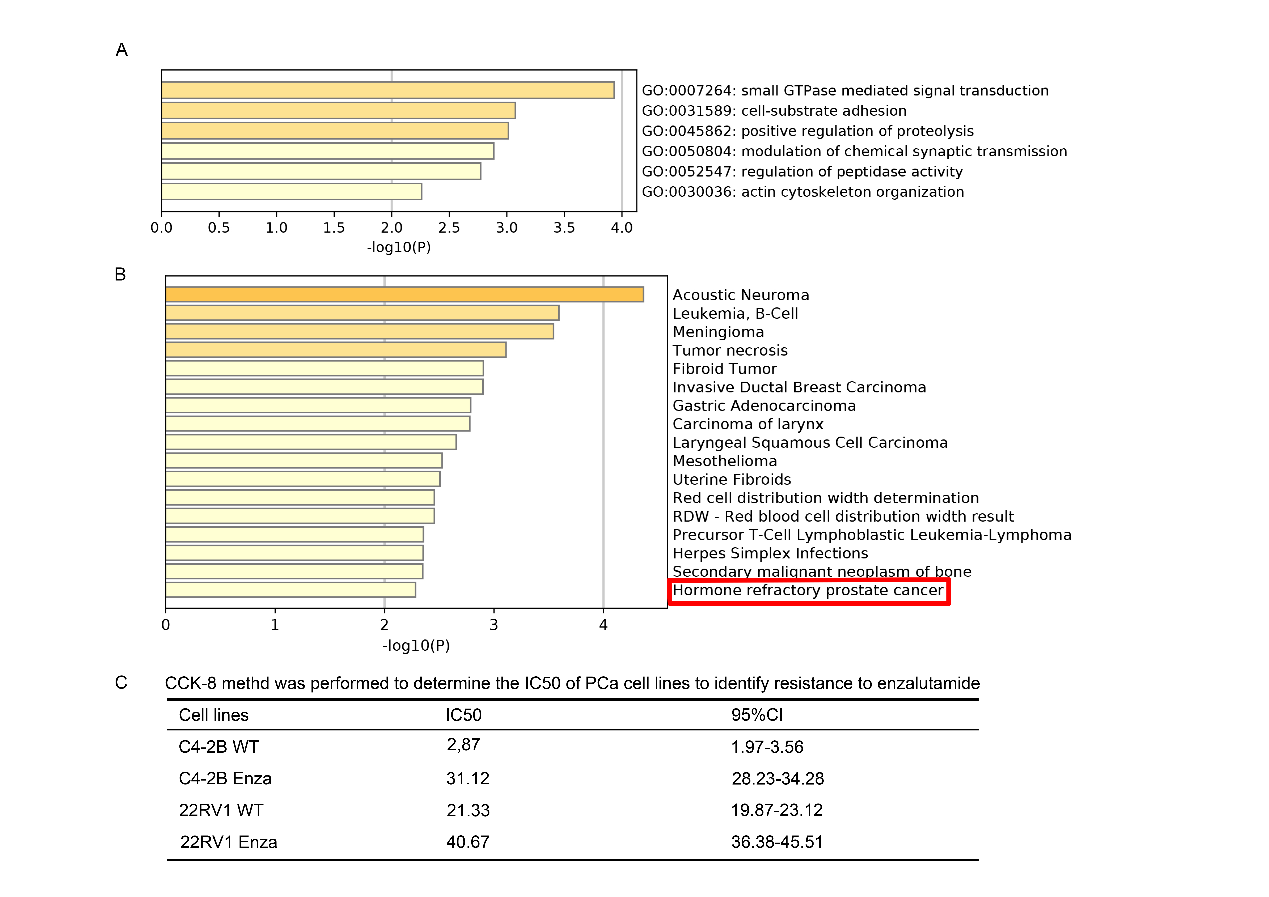


**Supplemental Figure 2**


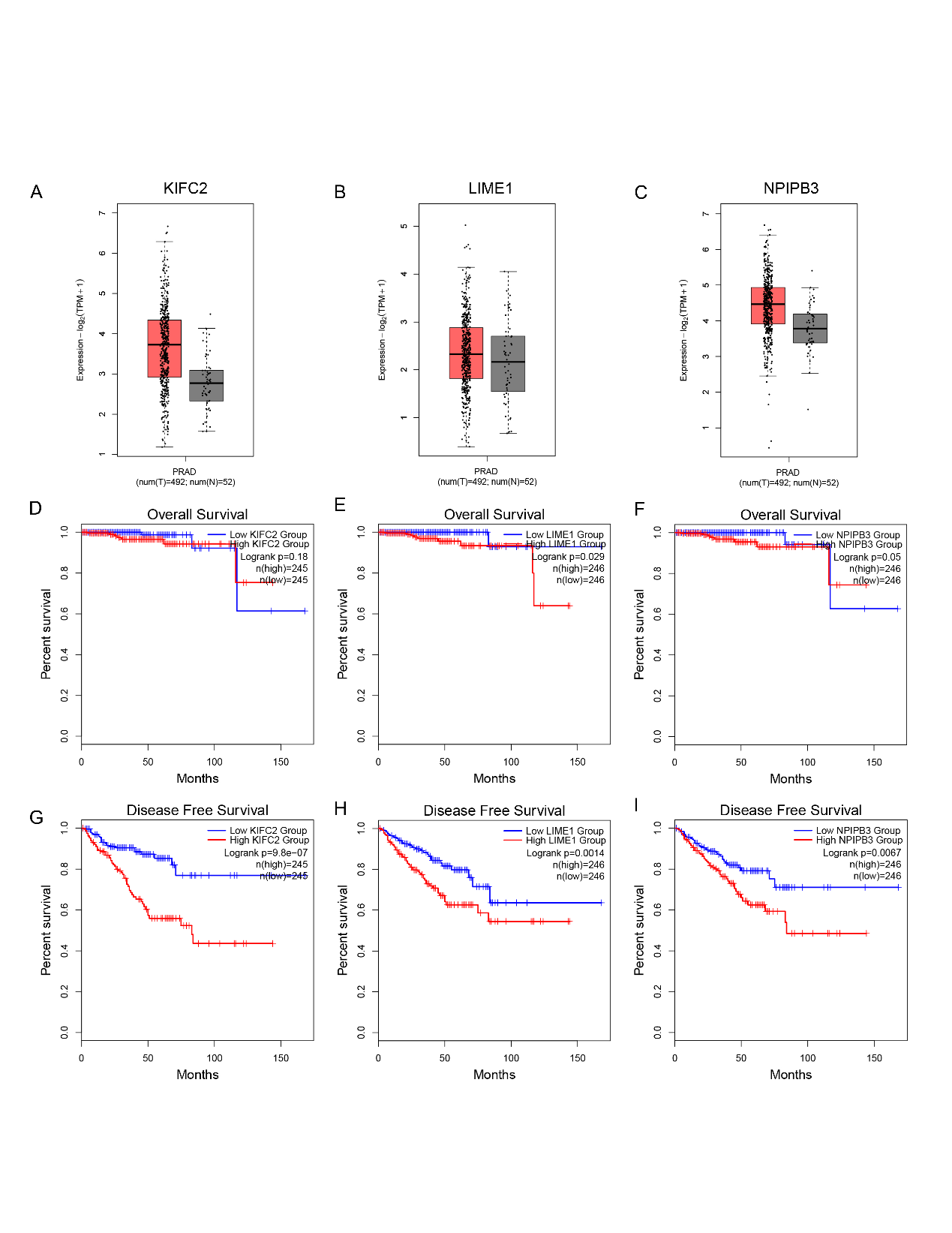


**Supplemental Figure 3**

**
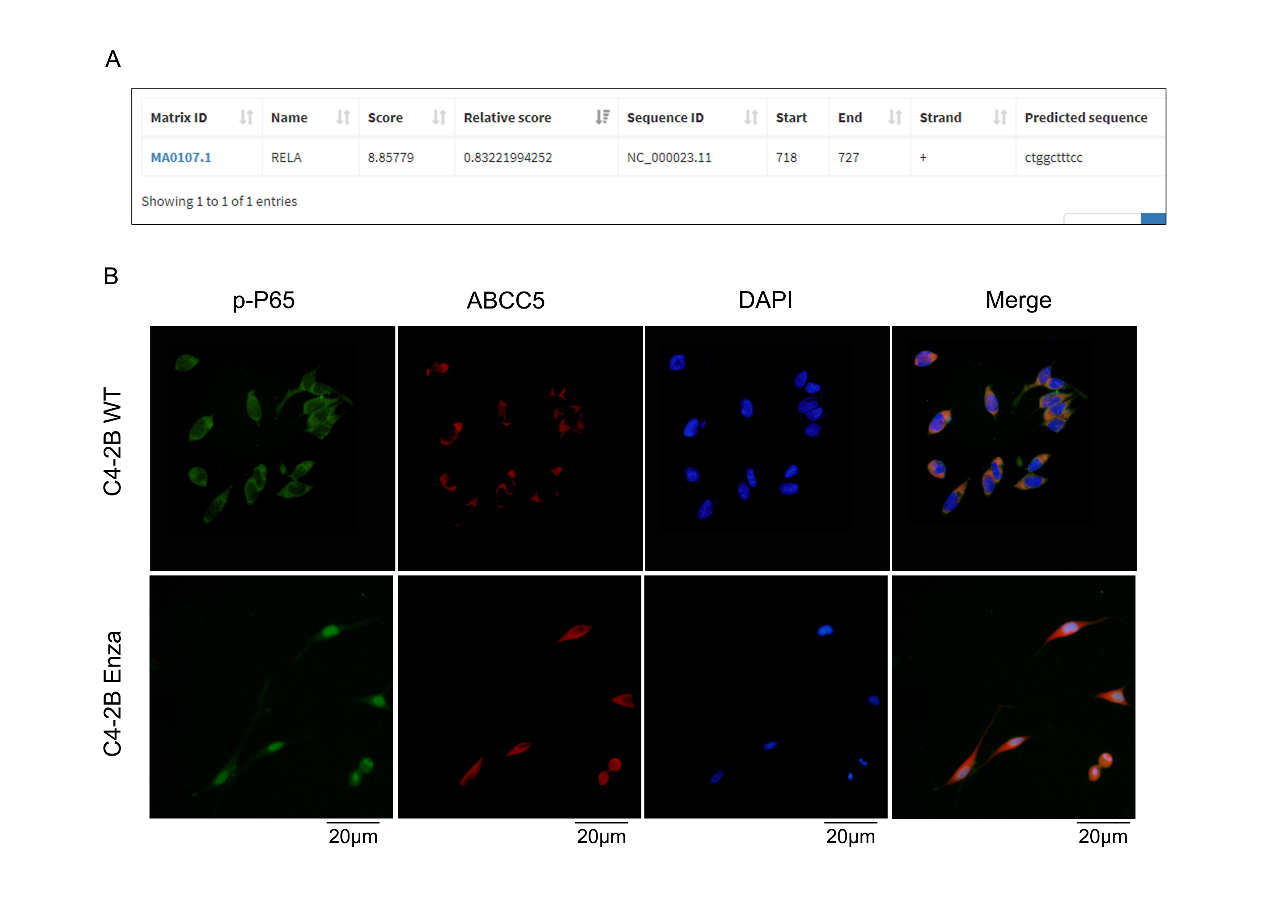
**

**Supplemental Figure 4

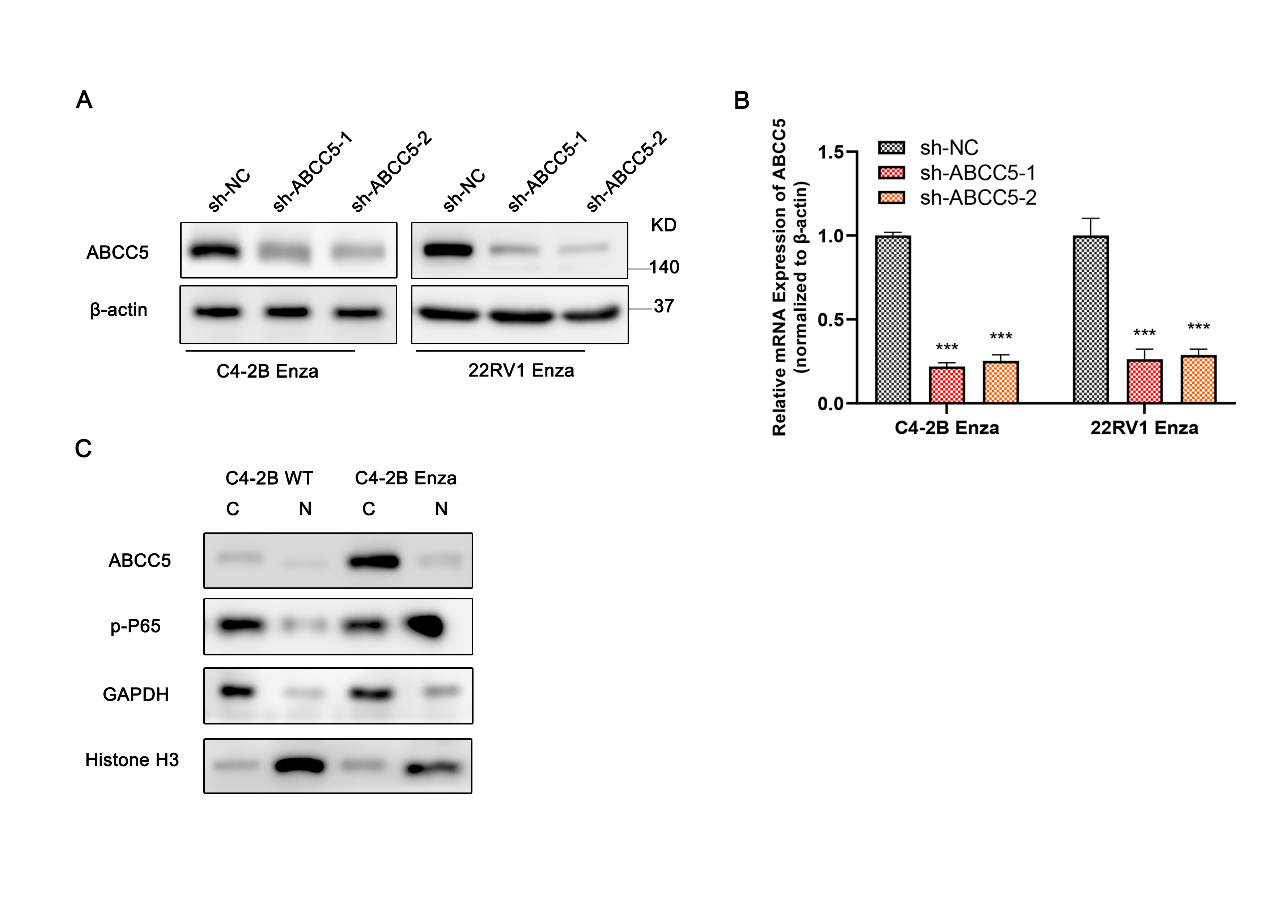
**

**Supplemental Figure 5

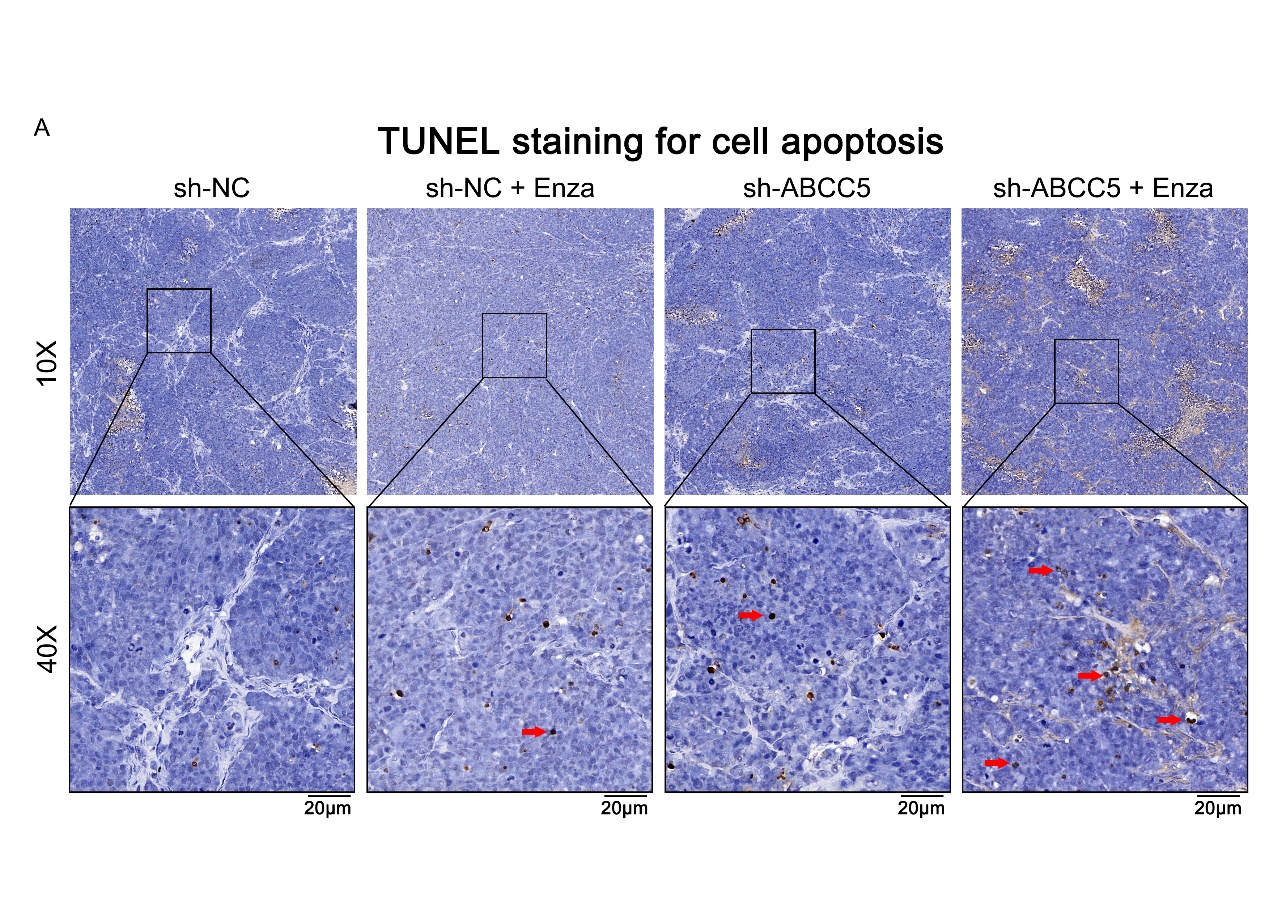
**

**plasmids information of pGL3.AR.WT and pGL3.AR.mutant**

**P65 binding region in pGL3.AR.WT promoter**

agatgtttatacaaatatttatagcagttttatttgtagtagccccaaactgaaaagaacccaaatgtccatcaaaagtgaatggataaacaaagcgtggtacagcaatgcaatagaatactacttagcaataaagaagaatgagctagtgatatacataacagcttaaatgtacatcaaaggcattgtgctcagtgaaagatgcaagtaaaaaaaaaaaagagtacatgctgtatagttccattgacataaaactctggaaagtgaaaaacagtctatactgacagaaagcagatcattggttgcctgaggaggaggagtataggagaggtggagggaaaatgtacaaagtggcacaataaaaacttttggaatcatagatatattcactatcttgattgagtgatgatttcatgagtgcacgcgtgtgtcaaaaatgatcaatttatgcaactttaaatatgtgcagtttattgtatatatcaattatacctcagtacggctattaaaaagaaaccctctggctgcacaatgcagaactgattctaggaaagagtggagggaggatgaccatttacagtgctccaggtggaagagaacggtgccttctggaagtgaactaggttggcaacaacagagatgaaataaatgggcagatgtgtgagatacttaggaaataaaacccgatggtcaccattttccaaaggtcagctcatc**ctggcttt**ccagagcaaagagctagggaagactttattaataaatccctcttgaagttgcagaggaagcttatagcagaaacttactctcaacctgactaatctgagagaacacctctggttccatttgattactaaaaaactgcaaagaacaggaggagaaagaagaagaaagctggtacaaacagtgaacttatataatattaatcaataattgtctcttgttcttaaaagcaatgggaagaaaatgagatttgagctggaagatcagagttcaaaatccaaataaagtatatggccctaatatgcttatagtagttaacctttcctgataatgatataattgttgacagcaccatctttaaaaataaaaataacatagtaatccttcagatttgtagaatgctttcctgtttacaagtttgttctatacacattatgtcttttaaatgacacactagccttctgagggtaacttatattggcaacagttttcagatgtggaaactgtgaagacaatgttggtgatgtggaagcaacataaactttggagtctttcagacccaggtttgaatgtcagactgctttttattcagagtaacttcagagcattatttctcaccttaattttttttcaggcctctttgtgtctatgtgtcctcttcactcctgtccattgttcattcagtgatttttgcaccttccttcactgttagtgtgtagacacatagttctcctggctctgagacctatgttaattccattctaccatcctgccagcccactcaattcctattgagcaatgctagttgaaagttgtggtgggattaaatgttgcaatgagtattcaaatgaggttgaagtatctacgcattctacttacatatggtgaggtatattcaaggaaggctgtagccattaaaatctcaggaaataatttttcacctcctcaggtgaaagggtcttcaggcctttgtgttctggaaggttcatttatagccatttcccaaatgacaatgcgattgatgagtctagagtctagctcaaatagcaatggactggaagactagtttaggttttactaatgtggaacatagaacaaattatgtccttgtttcagcctgttcatctgtgaaatagagcctatcatatccagtcttccttgcctttaggtttgagttaccttctttggtcaaggtaagtaaatgcctatgatgtttggctgtgcacaagataaagctacaacaaagctacaacccatcttttctctgtagaagactgcaa

**P65 binding region in pGL3.AR.mutant promoter**

agatgtttatacaaatatttatagcagttttatttgtagtagccccaaactgaaaagaacccaaatgtccatcaaaagtgaatggataaacaaagcgtggtacagcaatgcaatagaatactacttagcaataaagaagaatgagctagtgatatacataacagcttaaatgtacatcaaaggcattgtgctcagtgaaagatgcaagtaaaaaaaaaaaagagtacatgctgtatagttccattgacataaaactctggaaagtgaaaaacagtctatactgacagaaagcagatcattggttgcctgaggaggaggagtataggagaggtggagggaaaatgtacaaagtggcacaataaaaacttttggaatcatagatatattcactatcttgattgagtgatgatttcatgagtgcacgcgtgtgtcaaaaatgatcaatttatgcaactttaaatatgtgcagtttattgtatatatcaattatacctcagtacggctattaaaaagaaaccctctggctgcacaatgcagaactgattctaggaaagagtggagggaggatgaccatttacagtgctccaggtggaagagaacggtgccttctggaagtgaactaggttggcaacaacagagatgaaataaatgggcagatgtgtgagatacttaggaaataaaacccgatggtcaccattttccaaaggtcagctcatc**agttaggg**ccagagcaaagagctagggaagactttattaataaatccctcttgaagttgcagaggaagcttatagcagaaacttactctcaacctgactaatctgagagaacacctctggttccatttgattactaaaaaactgcaaagaacaggaggagaaagaagaagaaagctggtacaaacagtgaacttatataatattaatcaataattgtctcttgttcttaaaagcaatgggaagaaaatgagatttgagctggaagatcagagttcaaaatccaaataaagtatatggccctaatatgcttatagtagttaacctttcctgataatgatataattgttgacagcaccatctttaaaaataaaaataacatagtaatccttcagatttgtagaatgctttcctgtttacaagtttgttctatacacattatgtcttttaaatgacacactagccttctgagggtaacttatattggcaacagttttcagatgtggaaactgtgaagacaatgttggtgatgtggaagcaacataaactttggagtctttcagacccaggtttgaatgtcagactgctttttattcagagtaacttcagagcattatttctcaccttaattttttttcaggcctctttgtgtctatgtgtcctcttcactcctgtccattgttcattcagtgatttttgcaccttccttcactgttagtgtgtagacacatagttctcctggctctgagacctatgttaattccattctaccatcctgccagcccactcaattcctattgagcaatgctagttgaaagttgtggtgggattaaatgttgcaatgagtattcaaatgaggttgaagtatctacgcattctacttacatatggtgaggtatattcaaggaaggctgtagccattaaaatctcaggaaataatttttcacctcctcaggtgaaagggtcttcaggcctttgtgttctggaaggttcatttatagccatttcccaaatgacaatgcgattgatgagtctagagtctagctcaaatagcaatggactggaagactagtttaggttttactaatgtggaacatagaacaaattatgtccttgtttcagcctgttcatctgtgaaatagagcctatcatatccagtcttccttgcctttaggtttgagttaccttctttggtcaaggtaagtaaatgcctatgatgtttggctgtgcacaagataaagctacaacaaagctacaacccatcttttctctgtagaagactgcaa

**Supplemental table 1** Associations between ABCC5 expression and clinicopathological characteristics of PCa patients.

| **Clinical feature** | | | **Total patients n.** | | **Low n.** | | **High n.** | | **P-value (Low vs High)** |
| --- | --- | --- | --- | --- | --- | --- | --- | --- | --- |
| BPH | | | | 17 | | 14 | | 3 | **<0.01** |
| Primary Tumor | Gleason Score | ≤6 | | 10 | | 7 | | 3 | **<0.05** |
|  |  | 7-8 | | 31 | | 11 | | 20 | **<0.01** |
|  |  | >8 | | 16 | | 4 | | 12 | **<0.0001** |
| CRPC |  | ≤7 | | 3 | | 0 | | 3 | - |
|  |  | >7 | | 3 | | 0 | | 3 | - |

P-value < 0.05 was considered significant.

**Supplemental table 2** Characteristics of the corresponding antibodies included in the context.

| **Antibodies** | **Origin** | **SOURCE** | **IDENTIFIER** | **Application/Dilutions** |
| --- | --- | --- | --- | --- |
| ABCC5 | Rabbit | Abcam | ab180724 | IHC 1:50 IF 1:50 IB 1:1000 |
| AR | Rabbit | Abcam | ab133273 | IF 1:100 IB 1:2000 |
| AR-V7 | Rabbit | Abcam | ab198394 | IB 1:500 |
| PSA | Rabbit | Abcam | ab76113 | IB 1:1000 |
| β-actin | Rabbit | Servicebio | GB11001 | IB 1:1000 |
| Phospho-IKKα/β (Ser176/180) | Rabbit | CST | #2697 | IB 1:1000 |
| Phospho-IκBα (Ser32) | Rabbit | CST | #2859 | IB 1:1000 |
| Phospho-NF-κB p65(Ser536) | Rabbit | CST | #3033 | IB 1:1000 IF 1:200 |
| NF-kB p65 (acetyl K310) | Rabbit | Abcam | ab218533 | 2 µg for 25 µg of chromatin |
| Cleaved Caspase-3 | Rabbit | Servicebio | GB11532 | IF 1:300 |
| Ki-67 | Rabbit | Servicebio | GB111141 | IF 1:300 |
| GAPDH | Rabbit | Servicebio | GB11002 | IB 1:1000 |
| Histone H3 | Rabbit | Servicebio | GB11102 | IB 1:1000 |

**Supplemental Table 3. shRNA/siRNA list**

| shRNA/siRNA | Sequence (5’-3’) |
| --- | --- |
| sh*ABCC5*-1^1^ | CCGGCACCGCCAGTTGAGATCAATTCTCGAGAATTGATCTCAAC  TGGCGGTGTTTTTG |
| sh*ABCC5*-2 | CCGGTCTGTCGCCTTAGCATGTTTGCTCGAGCAAACATGCTAAG  GCGACAGATTTTTG |
| si*p65-*F^2^ | UCUUUCUGCACCUUGUCGCtt |
| si*p65-*R | GCGACAAGGUGCAGAAAGAtt |

**Supplemental Table 4. qPCR primer list**

| **qPCR Primer** | **Sequence (5’-3’)** |
| --- | --- |
| β-actin-F | TTCTGACCCATGCCCACCAT |
| β-actin-R | ATGGATGATATCGCCGCGCTC |
| PSA-F | GACCAAGTTCATGCTGTGTGC |
| PSA-R | CCACTCACCTTTCCCCTCAAG |
| SREBF1-F | GCCCCTGTAACGACCACTG |
| SREBF1-R | CAGCGAGTCTGCCTTGATG |
| ABCC5-F | GAAGAAAGATACAACTCTGTGCTG |
| ABCC5-R | GGATGTAGATGCTCCTGTCAC |

**Supplemental Table 5. PCR primer list**

| **ChIP PCR Primer** | **Sequence (5’-3’)** |
| --- | --- |
| AR-F | AAGAGTGGAGGGAGGATG |
| AR-R | TTGAGAGTAAGTTTCTGC |

**Reference**

1. Ji, G.*, et al.* Upregulation of ATP Binding Cassette Subfamily C Member 5 facilitates Prostate Cancer progression and Enzalutamide resistance via the CDK1-mediated AR Ser81 Phosphorylation Pathway. *Int J Biol Sci* **17**, 1613-1628 (2021).

2. Ganbold, T., Bao, Q., Zandan, J., Hasi, A. & Baigude, H. Modulation of Microglia Polarization through Silencing of NF-κB p65 by Functionalized Curdlan Nanoparticle-Mediated RNAi. *ACS Appl Mater Interfaces* **12**, 11363-11374 (2020).
