# Supplementary material for: Non-drug efflux function of ABCC5 promotes enzalutamide resistance in castration-resistant prostate cancer via upregulation of P65/AR-V7
Source: Cell Death Discov. 2022 May 3;8:241. doi: 10.1038/s41420-022-00951-4 (PMC9065095; doi:10.1038/s41420-022-00951-4)

Figure 2C

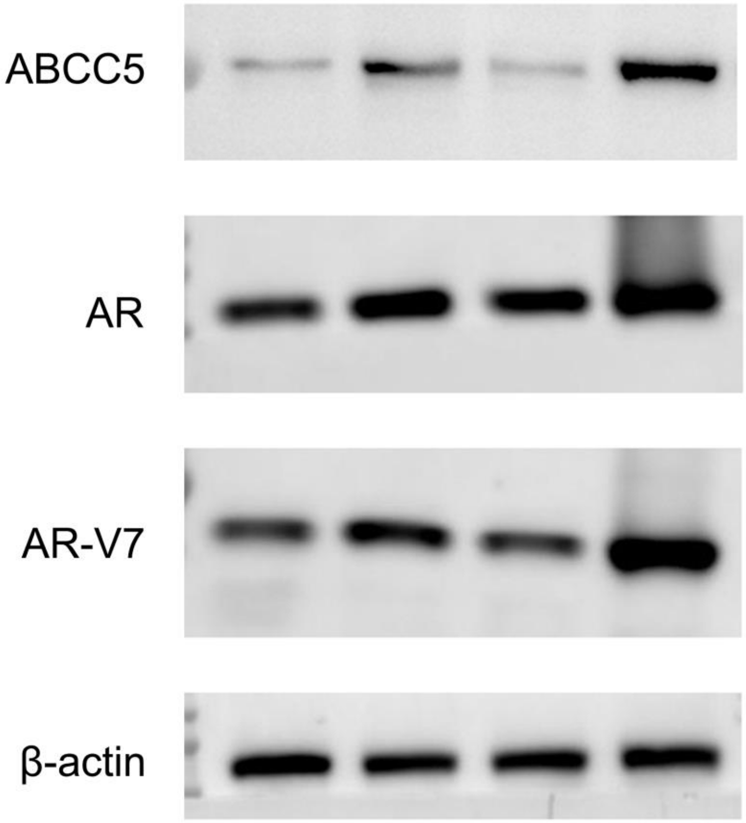

Figure 5A

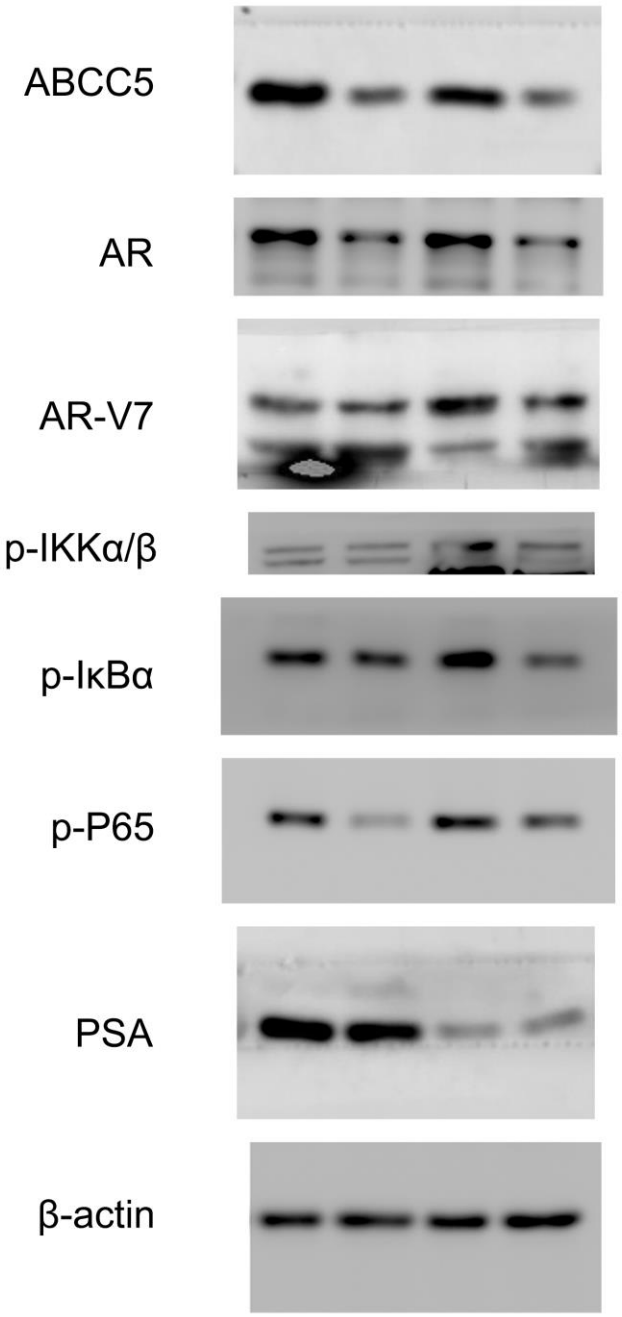

Figure 5B

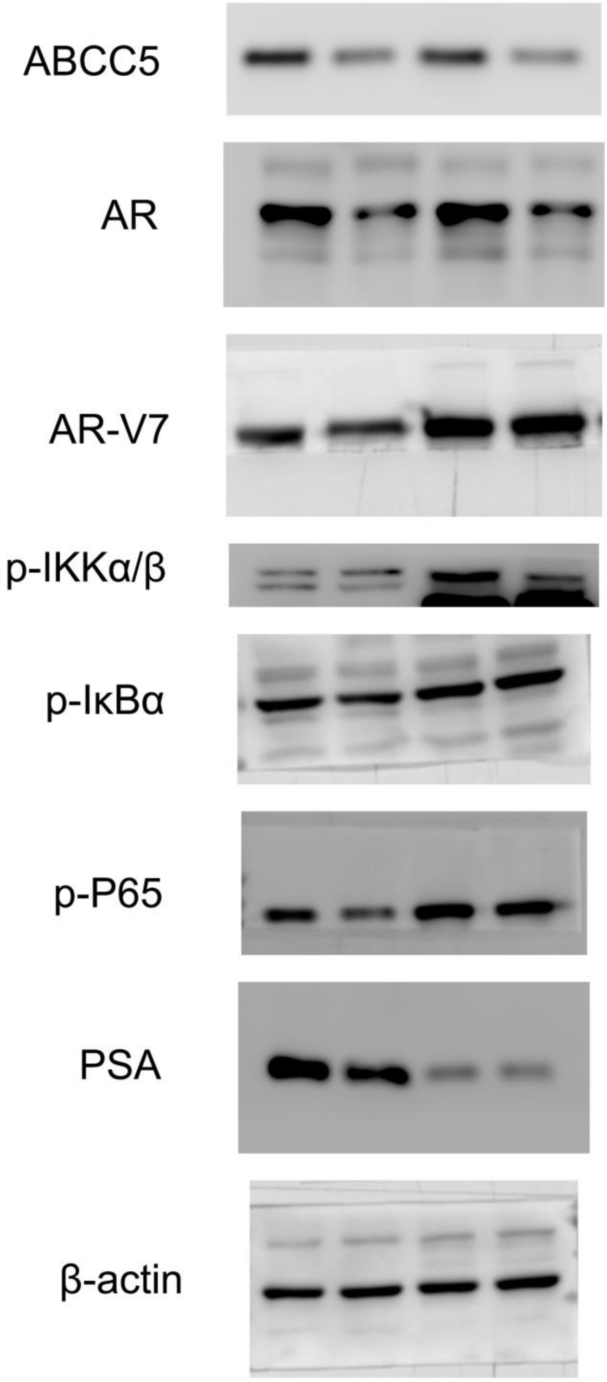

Figure 5C

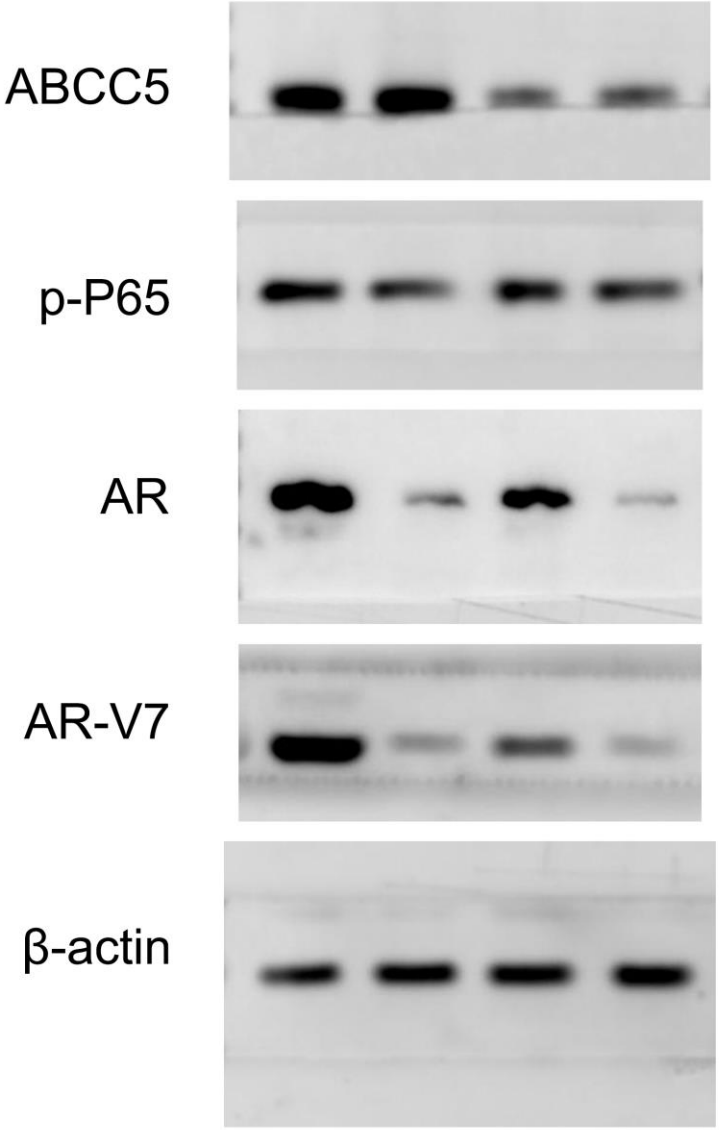

Figure 6B

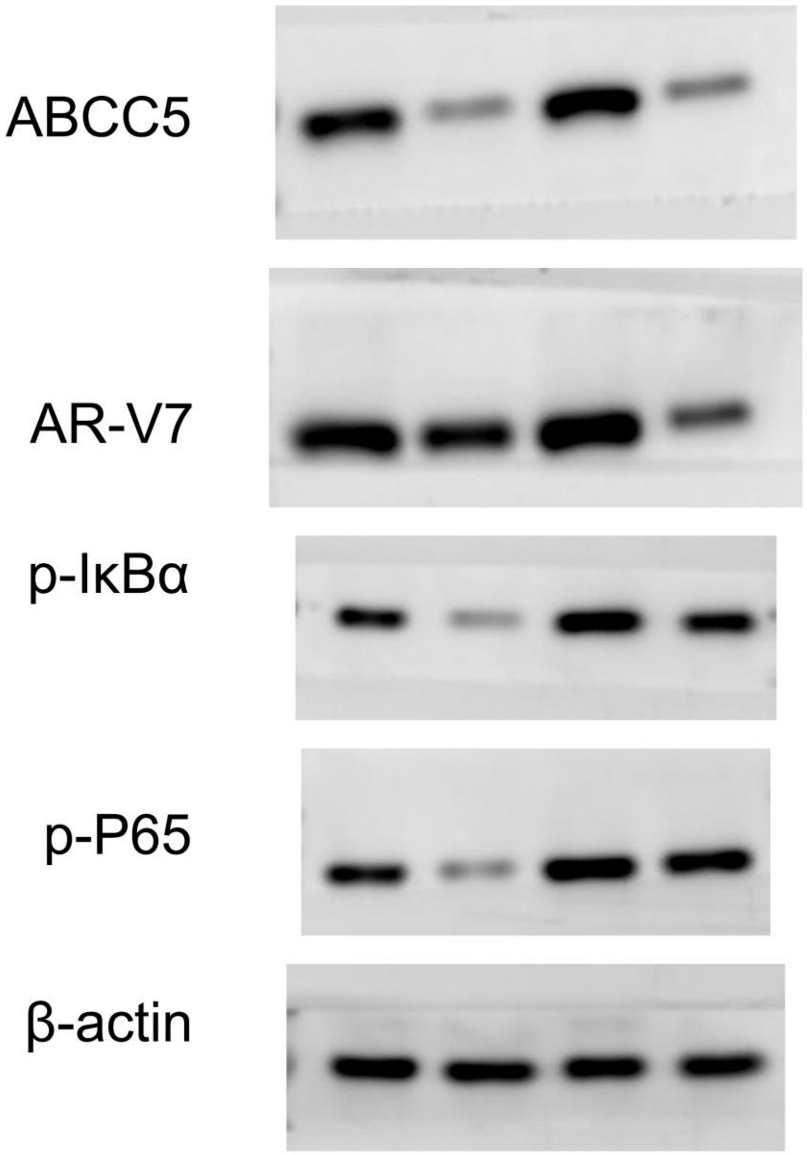

Figure 6C

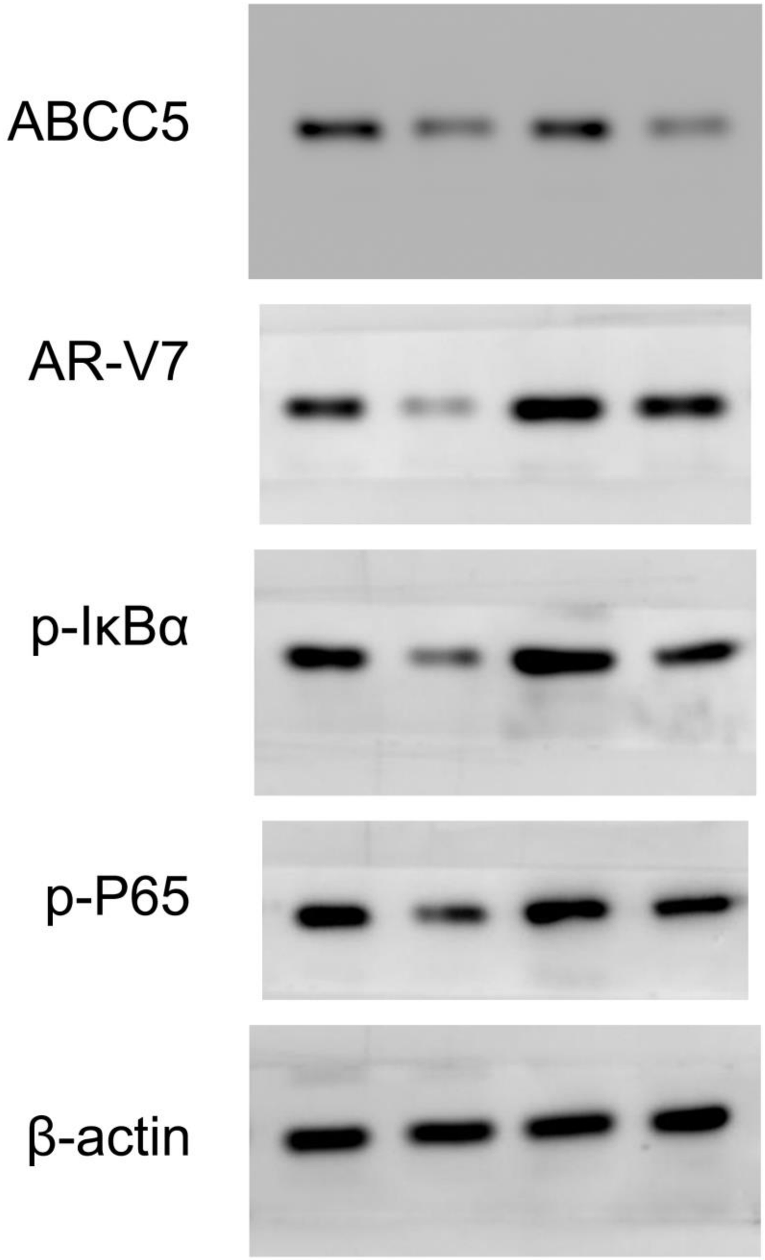

Supplemental Figure 4A

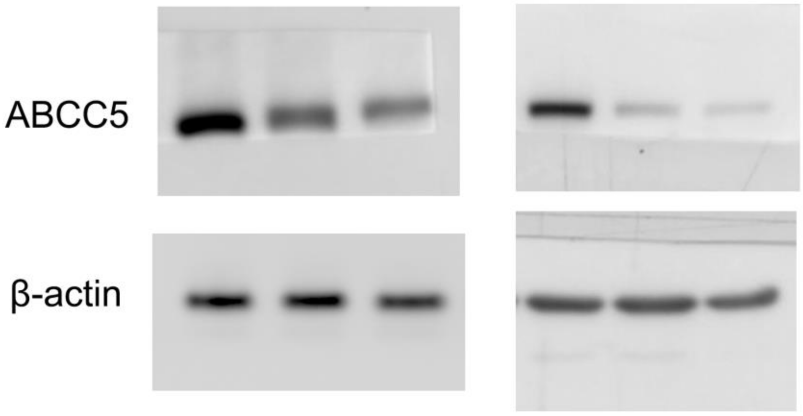

Supplemental Figure 4C

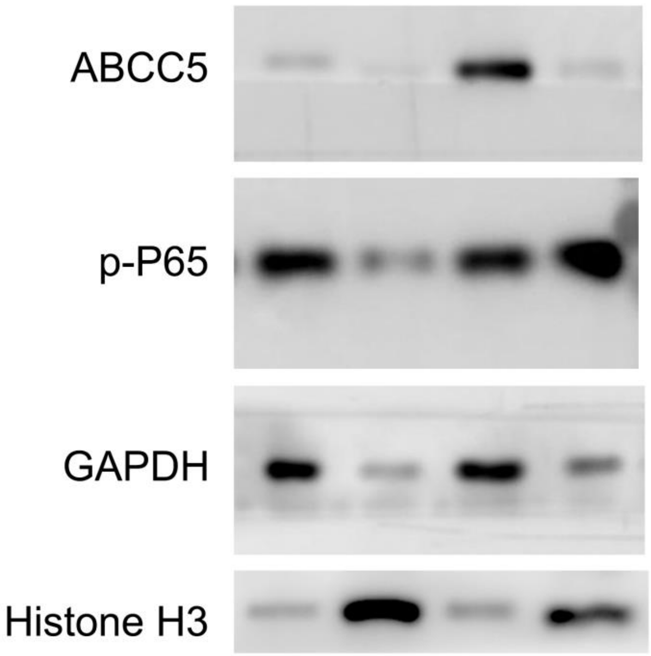

Supplement: Supplementary file 2 — Supplementary WB original scan [file 41420_2022_951_MOESM2_ESM.pdf]
